# A Novel Cofactor-binding Mode in Bacterial IMP Dehydrogenases Explains Inhibitor Selectivity*

**DOI:** 10.1074/jbc.M114.619767

**Published:** 2015-01-09

**Authors:** Magdalena Makowska-Grzyska, Youngchang Kim, Natalia Maltseva, Jerzy Osipiuk, Minyi Gu, Minjia Zhang, Kavitha Mandapati, Deviprasad R. Gollapalli, Suresh Kumar Gorla, Lizbeth Hedstrom, Andrzej Joachimiak

**Affiliations:** From the ‡ Center for Structural Genomics of Infectious Diseases,; ** Computational Institute, University of Chicago, Chicago, Illinois 60637,; the § Structural Biology Center, Biosciences, Argonne National Laboratory, Argonne, Illinois 60439, and; the Departments of ¶ Biology and; ‖ Chemistry, Brandeis University, Waltham, Massachusetts 024549110

**Keywords:** Antibiotic Resistance, Enzyme Inhibitor, Ligand-binding Protein, Microbial Pathogenesis, Nicotinamide Adenine Dinucleotide (NAD), Cryptosporidium parvum-selective Inhibitors, Antibacterial, Cofactor-binding Site, Inosine 5′-Monophosphate Dehydrogenase

## Abstract

The steadily rising frequency of emerging diseases and antibiotic resistance creates an urgent need for new drugs and targets. Inosine 5′-monophosphate dehydrogenase (IMP dehydrogenase or IMPDH) is a promising target for the development of new antimicrobial agents. IMPDH catalyzes the oxidation of IMP to XMP with the concomitant reduction of NAD^+^, which is the pivotal step in the biosynthesis of guanine nucleotides. Potent inhibitors of bacterial IMPDHs have been identified that bind in a structurally distinct pocket that is absent in eukaryotic IMPDHs. The physiological role of this pocket was not understood. Here, we report the structures of complexes with different classes of inhibitors of *Bacillus anthracis*, *Campylobacter jejuni,* and *Clostridium perfringens* IMPDHs. These structures in combination with inhibition studies provide important insights into the interactions that modulate selectivity and potency. We also present two structures of the *Vibrio cholerae* IMPDH in complex with IMP/NAD^+^ and XMP/NAD^+^. In both structures, the cofactor assumes a dramatically different conformation than reported previously for eukaryotic IMPDHs and other dehydrogenases, with the major change observed for the position of the NAD^+^ adenosine moiety. More importantly, this new NAD^+^-binding site involves the same pocket that is utilized by the inhibitors. Thus, the bacterial IMPDH-specific NAD^+^-binding mode helps to rationalize the conformation adopted by several classes of prokaryotic IMPDH inhibitors. These findings offer a potential strategy for further ligand optimization.

## Teaser

**Background:** IMP dehydrogenase (IMPDH) is an important drug target because of its role in *de novo* purine nucleotide biosynthesis.

**Results:** First substrate/cofactor- and substrate/inhibitor-bound complexes of bacterial IMPDHs are determined.

**Conclusion:** A new distinct binding mode of the cofactor adenosine moiety is revealed.

**Significance:** This work offers new insights for the design of more potent and selective inhibitors and the evolution of the active site.

## Introduction

Resistant pathogens are on the rise due to the overuse of antibiotics, poor hygiene, the increase of immunocompromised populations, and the ease of global travel. Multidrug-resistant and extensively drug-resistant *Mycobacterium tuberculosis*, methicillin-resistant and vancomycin-resistant *Staphylococcus aureus*, and bla_NDM-1+_/bla_KPC+_ *Klebsiella pneumoniae* pose worldwide threats (1, 2). The potential use of resistant pathogens in an act of bioterrorism creates another credible concern. Therefore, the discovery of new antibiotics that are effective against drug-resistant strains and the identification of new drug targets are of the highest urgency (3).

Inosine 5′-monophosphate dehydrogenase (IMPDH)^3^ is an emerging target for antibacterial drug discovery (4–9). IMPDH catalyzes the oxidation of inosine 5′-monophosphate (IMP) to xanthosine 5′-monophosphate (XMP) with the concurrent reduction of NAD^+^ to NADH. This reaction is the first and rate-limiting step in guanine nucleotide biosynthesis. The inhibition of IMPDH leads to the depletion of the guanine nucleotide pool, which blocks proliferation. IMPDH inhibitors are used as immunosuppressive, antiviral, and anticancer agents (10). Prokaryotic IMPDH-selective inhibitors could be a valuable addition to the existing pool of antibiotics.

The IMPDH reaction involves two chemical transformations. First, the catalytic Cys attacks IMP, and hydride is transferred to NAD^+^ to form the covalent intermediate E-XMP*. In the second step, E-XMP* is hydrolyzed to produce XMP. The enzyme has two essential but mutually exclusive conformations, an open conformation that accommodates both the substrate and cofactor during the dehydrogenase step, and a closed conformation where a mobile flap (referred to as the active site flap) moves into the cofactor-binding site for the hydrolysis of E-XMP* (10, 11). The dynamics of the IMPDH catalytic cycle makes the design of inhibitors more challenging because the structural consequences of inhibitor binding are difficult to predict.

**FIGURE 1.**
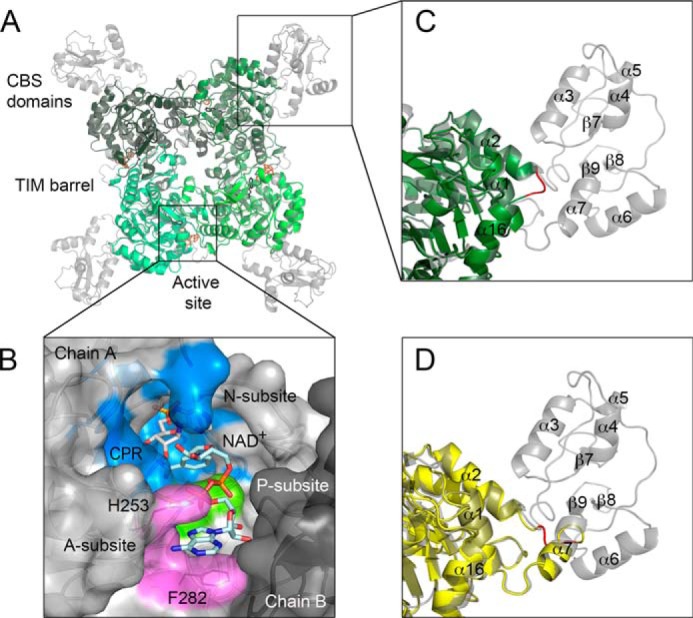
**Structures of wild type and CBS deletion mutants of IMPDH.** *A,* overlay of *Ba*IMPDH (*gray*) (11) and *Ba*IMPDHΔL·IMP·**2** (monomers are depicted in different shades of *green*). IMP (*black*) and **2** (*orange*) are shown as *sticks. B,* cofactor-binding site in human IMPDH2. The ternary complex of hIMPDH2 with NAD^+^ and a nonhydrolyzable substrate analog, CPR is shown (PDB code 1NFB). NAD^+^ binds in an extended conformation with the adenosine portion stacked between His-253 and Phe-282 (shown as *lines*). Chains A (*light gray*) and B (*dark gray*) are depicted in a surface representation. CPR (*white*) and NAD^+^ (*light teal*) are shown as *sticks*. Three NAD^+^ subsites, N-subsite (*blue*), P-subsite (*green*), and A-subsite (*violet*) are represented. *C,* zoom of the same overlay as in *A*, showing the SGG connecting sequence (*red*) in *Ba*IMPDHΔL·IMP·**2**. Color code as in *A*. Secondary structure elements are also labeled. *D,* zoom of the overlay of wild type *Ba*IMPDH (*gray*) and *Ba*IMPDHΔS (*yellow*). ΔS constructs are longer than ΔL variants by ∼20 residues. Some of these residues make up α-helix 7. SGG connecting sequence is shown in *red*.

IMPDHs are tetramers with a D4 square symmetry (Fig. 1*A*). Monomers are composed of two domains, the catalytic domain with a classic (β/α)_8_ barrel fold (also known as a TIM-barrel) (12), and a smaller domain containing tandem cystathione β-synthetase (CBS) motifs (10, 13). The CBS domain is inserted in a distal α2/β3 loop of the barrel domain (14) and protrudes away from the corners of the tetramer (Fig. 1*A*). This domain is often the least ordered part of an IMPDH structure. The physiological role of the CBS domain is not understood, and deletion of the CBS domain has little or no effect on IMPDH catalytic activity or tetramer formation (15–17).

A substrate- and cofactor-bound structure of a bacterial IMPDH has not been reported before. All available structures of NAD^+^/NAD-analog complexes deposited to date are of eukaryotic enzymes. In these structures, the IMP and cofactor-binding sites are located near the subunit interface, between two (β/α)_8_ barrel domains, with the majority of protein-ligand contacts occurring within the same monomer (1NF7 and 1NFB; Fig. 1*B*) (18–20). Structural and similarity comparisons indicate that the IMP site is well defined and highly conserved. In contrast, the cofactor site is more diverged among IMPDHs, and this site has been targeted for the development of species-specific inhibitors (6, 10, 21). Despite the variability of the cofactor-binding site, NAD^+^ was generally assumed to bind in a similar mode in both bacterial and eukaryotic enzymes.

For the purpose of inhibitor design, the NAD^+^-binding site is subdivided into three subsites as follows: the nicotinamide riboside-binding site (N-subsite); the pyrophosphate-binding site (P-subsite); and the adenosine-binding site (A-subsite) (Fig. 1*B*). The N-subsite is conserved, as expected given that it is the site of chemical transformation. It is more difficult to demarcate boundaries for the P- and A-subsites because the sequences of these sites are more diverged, and the structures of proteins are often disordered in this region. The variability and flexibility of the A-subsite make predictions of the inhibitor-binding mode especially challenging, underlining the importance of obtaining crystal structures of IMPDHs in complex with different classes of inhibitors.

**FIGURE 2.**
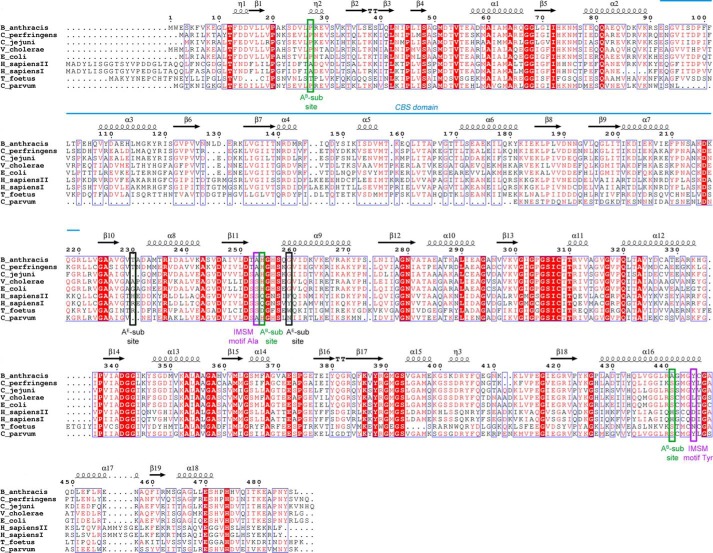
**Multiple sequence alignment of selected bacterial and eukaryotic IMPDHs.** Identical residues are highlighted in *red,* and similar residues are shown as *red letters*. Secondary structure elements derived from *Ba*IMPDH (PDB code 3TSB (11)) are depicted as *arrows* (representing β-strands) and *coils* (representing α- and 3_10_-helices). The location of tandem CBS domains is shown as a *blue line*. Positions of two residues forming the IMSM are marked as *purple rectangles*. Positions of residues involved in binding of the NAD^+^ adenosine moiety in bacterial (A^B^-subsite) and eukaryotic (A^E^-subsite) enzymes are indicated by *green* and *black rectangles*, respectively. The sequences used in the alignment include *B. anthracis* str. Ames (gi: 30253523), *C. perfringens* (gi: 110800169), *C. jejuni* subsp. *jejuni* (gi: 15792385), *V. cholera* O1 biovar (gi: 15640786), *E. coli* str. K-12 (gi: 388478544), *H. sapiens* I (gi: 217035148) and *H. sapiens* II (gi: 66933016), *T. foetus* (gi:28373644), and *C. parvum* (gi: 323510309). The alignment was generated using MultiAlin (53) and ESPript (54) programs.

Although eukaryotic and prokaryotic IMPDHs have similar overall folds, they differ significantly in their structural details, kinetic properties, and sensitivity to inhibitors (10, 22, 23). Prokaryotic IMPDH-specific inhibitors were initially discovered in a high throughput screen for NAD^+^ site inhibitors of *Cryptosporidium parvum* IMPDH (*Cp*IMPDH) (24), a parasite that has a bacterium-like IMPDH. Further medicinal chemistry optimization has produced compounds with high potency and selectivity *versus* human IMPDHs in several different chemical scaffolds (designated as classes A, C, D, P, and Q, among others) (25–30). Structural characterization of *Cp*IMPDH with inhibitors C64 and Q21 indicated that these compounds bind in a different site than the one observed for NAD^+^ in eukaryotic IMPDHs. These structures revealed an “inhibitor minimal structural motif” (IMSM) of Ala-165 and Tyr-358′ (prime denotes a residue from the adjacent monomer) that accounts for inhibitor selectivity *versus* human enzymes (Fig. 2) (5, 11, 28, 31). This motif is found in IMPDHs from many important bacterial pathogens, including *M. tuberculosis*, *S. aureus*, *K. pneumoniae, Bacillus anthracis*, *Helicobacter pylori*, *Streptococcus pyogenes*, *Clostridium perfringens,* and *Campylobacter jejuni* but, interestingly, not *Vibrio cholerae* (5). Many *Cp*IMPDH inhibitors are also potent inhibitors of *B. anthracis* IMPDH, and several display significant antibacterial activity against *B. anthracis* and other Gram-positive bacteria (9).

**FIGURE 3.**
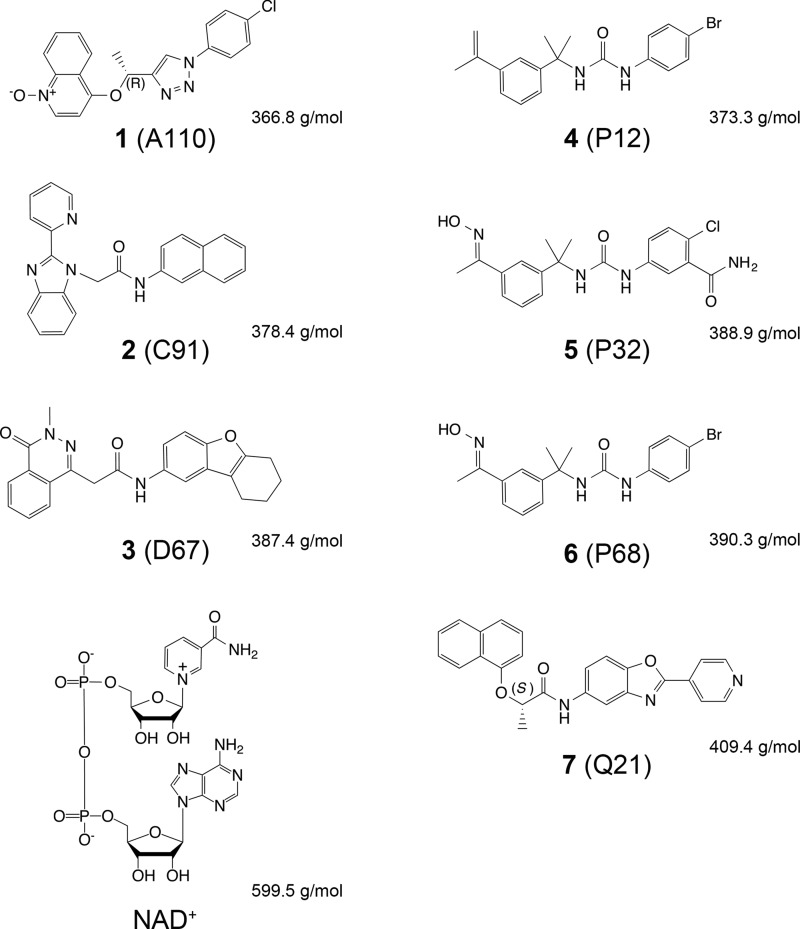
***Cp*IMPDH-selective inhibitors tested in this study.** The code in parentheses consists of a letter that refers to the class of compounds (A, C, D, P, and Q) discovered during the high throughput screen against *Cp*IMPDH (24) and a number that indicates a particular class representative. Stereochemistry is indicated where applicable. NAD^+^ is shown for comparison. Molecular weights of compounds are also listed.

IMPDHs from four bacterial pathogens were chosen to investigate the spectrum of inhibition of *Cp*IMPDH-specific inhibitors. IMPDHs from *B. anthracis* (*Ba*IMPDH), *C. jejuni* (*Cj*IMPDH), and *C. perfringens* (*Clp*IMPDH) possess the IMSM and therefore should all be sensitive to the *Cp*IMPDH-specific inhibitors. In contrast, IMPDH from *V. cholerae* (*Vc*IMPDH) lacks the IMSM and was shown to be resistant to these compounds (11). Here, we present x-ray crystal structures of CBS deletion variants of *Ba*IMPDH, *Cj*IMPDH, and *Clp*IMPDH with inhibitor **2** (Fig. 3). We also determine the structures of the complexes of compound **1** with *Ba*IMPDH and *Clp*IMPDH, of compound **4** with *Clp*IMPDH and of four additional inhibitors with *Ba*IMPDH (**3**, **5**, **6,** and **7**) (Fig. 3). Comparison of these structures provides the basis for inhibitor selectivity and offers a potential strategy for further optimization. Moreover, we report two structures of *Vc*IMPDH in complex with NAD^+^. One structure contains the cofactor and a mixture of IMP and covalent intermediate in the active site, representing the IMPDH reaction in progress. The second is a high resolution structure containing the product XMP and NAD^+^ and thus corresponds to the final stage of the reaction. These structures reveal a dramatically different mode of NAD^+^ binding than the one observed for eukaryotic IMPDHs and help to rationalize the binding mode adopted by several classes of inhibitors.

## EXPERIMENTAL PROCEDURES

### 

#### 

##### Materials

IMP was purchased from Acros Organics (currently Thermo Fisher Scientific). XMP was purchased from Fluka. NAD^+^ was purchased from Sigma. The following seven *C. parvum*-selective inhibitors (Fig. 3) were synthesized as described in Refs. 5, 26, 29, 30: **1** (4-[(1*R*)-[1-(4-chlorophenyl)-1*H*-1,2,3-triazol-4yl]ethoxy]quinoline-1-oxide, C_19_H_15_ClN_4_O_2_); **2** ((α-methyl-*N*-2-naphthalenyl-2-(2-pyridinyl)-1*H*-benzimidazole-1-acetamide, C_24_H_18_N_4_O]); **3** (3,4-dihydro-3-methyl-4-oxo-*N*-(6,7,8,9-tetrahydro2-dibenzofuranyl)-1-phthalazineacetamide, C_23_H_21_N_3_O_3_); **4** (*N*-(4-bromophenyl)-*N*-[1-methylethenyl)phenyl]ethyl urea, C_19_H_21_BrN_2_O); **5** (2-chloro-*N*-methyl-5-[[[[1-methyl-1-[3-(1-methylethenyl)phenyl]ethyl]amino]carbonyl]aminobenzamide,C_19_H_21_ClN_4_O_3_) (30); **6** (*N*-(4-bromophenyl)-*N*-[1-[3-[1-(hydroxyimino)ethyl]phenyl]-1-methylethyl urea, C_18_H_20_BrN_3_O_2_); and **7** (2-(1-naphthalenyloxy)-*N*-[2-(4-pyridinyl)-5-benzoxazolyl)-(2*S*)-propanamide, C_25_H_19_N_3_O_3_) (28).

##### Generation of CBS Domain Deletion Constructs

Two CBS deletion constructs (short (ΔS) and long (ΔL) deletion) were prepared for each protein using crystal structures of bacterial IMPDHs as a guide. The ΔL construct involved the removal of ∼120 residues, and the ΔS construct involved deletion of ∼100 residues (Fig. 1, *C* and *D*, and Table 1). In both cases, the removed portion was replaced with a three-amino acid connecting sequence (SGG). For example, in the case of *Ba*IMPDH, the ΔL included Glu-92–Arg-220 and the ΔS Val-95–Thr-200 residues, respectively. The deleted sequence was replaced with GG or G for the ΔL and ΔS, respectively, to create an SGG connector. Using the *Ba*IMPDH gene in vector pMCSG7 (11) as a template, codons for residues Glu-92–Arg-220 (ΔL) or Val-95–Thr-200 (ΔS) were replaced with codons for GG (ΔL) or G (ΔS) by the megaprimer cloning method (32) with some modifications. First, the Glu-92–Arg-220 deletion primer and the *Ba*IMPDH coding sequence forward primer were used to amplify a region of *Ba*IMPDH-pMCSG7 ranging from residue Met-1 to residue Val-229 with a 5′-LIC overhang, while replacing codons for residues Glu-92–Arg-220 with codons for GG. The resulting product was used as a megaprimer in the whole plasmid synthesis reaction, with *Ba*IMPDH-pMCSG7 as a template and the reverse primer encoding the 3′-end of *Ba*IMPDH (Table 2). PCRs contained KOD Hot Start DNA polymerase kit (Novagen, Madison, WI). Cycling was performed at 95 °C for 3 min, followed by 95 °C for 40 s, 53 °C for 40 s, and 72 °C for 1.5 min for 32 cycles. The resulting product was treated with T4 polymerase, annealed into the pMCSG7 vector, and transformed into BL21(DE3)/pMAGIC cells. All of the resulting clones were sequenced to verify mutations. Similar procedures were followed to construct the ΔS and ΔL mutants for *Cj*IMPDH and *Clp*IMPDH. Primer sequences are listed in Table 2.

**TABLE 1:** **CBS deletion mutant characteristics**

Species	Deletion type	Deleted residues	Inserted residue(s)*^a^*	Protein size	Label
				*Da*	
*B. anthracis*	Short	Val-95–Thr-200	G	40,435	*Ba*IMPDHΔS
*B. anthracis*	Long	Glu-92–Arg-220	GG	37,920	*Ba*IMPDHΔL
*C. jejuni*	Short	Val-92–Thr-195	G	40,722	*Cj*IMPDHΔS
*C. perfringens*	Long	Gln-89–Arg-215	SGG	38,123	*Clp*IMPDHΔL
*V. cholerae*	Long	Phe-91–Arg-219	SGG	37,929	*Vc*IMPDHΔL

*^a^* Single letter amino acid codes are used.

**TABLE 2:** **Sequences of primers used to prepare IMPDH ΔCBS mutants** A set of three primers was utilized for each construct, where F, Del R, and R designate forward, deletion reverse, and reverse primer, respectively. Inserted connecting sequence (resulting in G, GG, or SGG amino acid sequence) in deletion reverse primer is shown in bold type. NA, not applicable.

Construct	Primer direction	Deleted residue(s)	Inserted residue(s)	PCR primer sequence 5′to 3′
*Ba*IMPDHΔS	F	NA	NA	TACTTCCAATCCAATGCCATGTGGGAATCTAAATTTGTTAAAGAAGGTCT
*Ba*IMPDHΔS	Del R	Val-95–Thr-200	Gly	GAATTTGGGAATTCAATTACTTTTTCAATATCTTTTAT**ACC**GCCACTTTCAGAACGTTTTACTTTATCAA
*Ba*IMPDHΔS	R	NA	NA	TTATCCACTTCCAATGTTATTATAATGAGTAGTTTGGAGCCTCTTTTGTAATTT
*Ba*IMPDHΔL	F	NA	NA	TACTTCCAATCCAATGCCATGTGGGAATCTAAATTTGTTAAAGAAGGTCT
*Ba*IMPDHΔL	Del R	Glu-92–Arg-220	GG	ACACCAACTGCTGCTCCAACTAATAA**ACCACC**AGAACGTTTTACTTTATCAACTTGCTCGG
*Ba*IMPDHΔL	R	NA	NA	TTATCCACTTCCAATGTTATTATAATGAGTAGTTTGGAGCCTCTTTTGTAATTT
*Cj*IMPDHΔS	F	NA	NA	TACTTCCAATCCAATGCCATGAAAATTGTAAAAAGAGCTTTAACTTTTGAAGATGTAT
*Cj*IMPDHΔS	Del R	Val-92–Thr-195	Gly	ATCAGGATATTCTTTGCGTTTTTTAAGATCTTTTAT**ACC**CCCGCTTTCACTTTTTTTCACTCTTTTTA
*Cj*IMPDHΔS	R	NA	NA	TTATCCACTTCCAATGTTATCATTGATGATTGACCTTGTAATTTGGTG
*Clp*IMPDHΔL	F	NA	NA	TACTTCCAATCCAATGCCATGGCAAGAATATTAAAAACAGCATATACATTTGAT
*Clp*IMPDHΔL	Del R	Gln-89–Arg-215	SGG	CACCGATTGAAGCTCCACATAAAAG**ACCACCACT**TCTTTTTACTCTATCAACTTCTCTTGCTTGAT
*Clp*IMPDHΔL	R	NA	NA	TTATCCACTTCCAATGTTATTATTGGTTAACACTGTAGTTTGGTGCTT
*Vc*IMPDHΔL	F	NA	NA	TACTTCCAATCCAATGCCTTGCACATGCTACGAATCGCAAAAGAA
*Vc*IMPDHΔL	Del R	Phe-91–Arg-219	SGG	AGCGGCACCAACACGCAG**ACCACCACT**GATTTTCACTTGGTGAACTTGAGCC
*Vc*IMPDHΔL	R	NA	NA	TTATCCACTTCCAATGTTATTAACCCAGACGGTAGTTTGGTGC

##### Protein Expression, Purification, and Crystallization

All IMPDHs were expressed according to a standard protocol (11, 33). All proteins were appended to an N-terminal His_6_ tag and were purified using nickel(II) affinity chromatography (IMAC). For *Clp*IMPDHΔL and *Vc*IMPDHΔL enzymes, the His_6_ tag was subsequently removed with TEV protease, leaving the vector-derived SNA sequence at the N terminus, and the His_6_ tag-free protein was additionally purified using a subtractive IMAC to remove the released tag and uncut protein. The proteins were then dialyzed into crystallization buffer containing 20 mm HEPES, pH 8.0, 150 mm KCl, and 2 mm DTT or 1.5 mm TCEP. Because the His_6_ tag could not be removed in the case of *Ba*IMPDHΔS, *Ba*IMPDHΔL, and *Cj*IMPDHΔS, the purification procedure of the His_6_ tag-appended IMPDHs consisted of one IMAC step followed by a size exclusion chromatography step on a HiLoad Superdex 200 16/600 column (GE Healthcare).

For each protein, crystallization screening was set up with the help of a Mosquito liquid dispenser (TTP LabTech, Cambridge, MA) using the sitting-drop, vapor-diffusion method in 96-well CrystalQuick plates (Greiner Bio-One, Monroe, NC). For co-crystallization trials, IMP and inhibitors were used at 10- and 4–10-fold molar excess, respectively, over protein concentration. For each condition, 0.4 μl of protein solution and 0.4 μl of crystallization formulation were mixed, and the mixture was equilibrated against a 135-μl reservoir. The suite of INDEX (Hampton Research, Aliso Viejo, CA), four MCSG crystallization screens (Microlytic, Woburn, MA), and Pi-minimal (Jena Bioscience GmbH, Jena, Germany) were used, and conditions yielding diffraction quality crystals typically appeared within 2–7 days. Crystallization conditions for inhibitor complexes are listed in Tables 3 and 4. Crystals of the *Vc*IMPDHΔL·IMP·NAD^+^ complex were obtained by soaking *Vc*IMPDHΔL·IMP crystals with 200 mm aqueous solution of NAD^+^ for 15 min at 20 °C and followed by cryo-protection. Crystals of *Vc*IMPDHΔL·XMP·NAD^+^ complex were obtained following the same procedure, but incubation of the soaked crystals was carried out for 5 days. Crystallization conditions for *Vc*IMPDHΔL complexes are listed in Table 5.

**TABLE 3:** **Crystallization conditions, data collection, and refinement statistics** ASU indicates asymmetric unit.

	*Ba*IMPDHΔS	*Ba*IMPDHΔL·IMP·1	*Ba*IMPDHΔL·IMP·2	*Ba*IMPDHΔL·IMP·3	*Ba*IMPDHΔL·IMP·5	*Ba*IMPDHΔL·IMP·6
**Data collection**						
Space group	*P*1	*P*4	*P*1	*P*21	*P*1	*P*1
Cell dimensions						
*a, b, c* (Å)	84.33, 84.25, 84.31	110.85, 110.85, 56.26	84.93, 89.88, 104.62	83.13, 101.33, 87.27	85.37, 89.82, 104.50	83.22, 89.39, 103.99
α, β, γ (°)	110.01, 109.22, 109.19	90.00, 90.00, 90.00	98.70, 90.32, 96.46	90.00, 109.57, 90.00	81.41, 90.42, 83.50	81.30, 89.95, 83.59
Protein molecules/ASU	4	2	8	4	8	8
Wavelength (Å)	0.9792	0.9792	0.9792	0.9793	0.9792	0.9793
Resolution (Å)*^a^*	2.25 (2.25-2.24)	1.90 (1.90-1.93)	2.60 (2.60-2.64)	2.80 (2.80-2.85)	2.70 (2.70-2.75)	2.60 (2.60-2.64)
Unique reflections	79311 (4081)	53,268 (2224)	91,462(4515)	33,129 (1545)	82,135 (4094)	88,256 (4394)
*R*_merge_ *^b^*	0.052 (0.469)	0.090 (0.463)	0.081(0.407)	0.154 (0.749)	0.125 (0.584)	0.090 (0.426)
〈*I/*σ*I*〉	12.7 (2.7)	7.5 (1.7)	11.1(2.2)	7.4 (1.6)	6.4 (1.9)	8.4 (2.2)
Completeness (%)	94.5 (96.2)	98.3 (81.6)	98.1(97.1)	98.4 (93.5)	98.4 (98.0)	97.8 (97.8)
Redundancy	2.2 (2.1)	4.3 (2.0)	1.9 (1.9)	3.6 (3.1)	2.3 (2.0)	2.2 (2.2)

**Refinement**						
Resolution (Å)	2.25 (2.25-2.29)	1.90 (1.90-1.93)	2.60(2.60-2.62)	2.80 (2.80-2.88)	2.70 (2.70-2.73)	2.60 (2.60-2.63)
Reflections: work/test set	75225/4055	50,550/2697	86,761/4583	31,341/1681	77,888/4096	83,785/4415
*R*_work_/*R*_free_*^c^*	0.206 (0.232)	0.147 (0.184)	0.170 (0.216)	0.184 (0.248)	0.218 (0.260)	0.194 (0.253)
No. of atoms: protein/ligands*^d^*						
Water	9632/32/464	5119/116/491	20473/479/310	9854/208/17	20811/526/403	20517/376/117
Average *B* factor (Å^2^): protein/ligand(s) water	49.5/50.8/41.0	23.4/23.6/30.9	45.5/46.9/33.6	68.9/65.4/54.7	47.3/46.9/34.8	60.7/55.5/48.6
Bond lengths (Å)	0.004	0.007	0.003	0.011	0.002	0.002
Bond angles (°)	0.854	1.216	0.740	1.397	0.659	0.649
Most favored	92.2	96.4	96.5	97.4	95.4	95.44
Outliers	0.08	0.31	0.33	0.00	0.48	0.36
PDB code	4MJM	4MYA	4MY9	4QM1	4MYX	4MY1
Crystallization conditions	0.2 m sodium chloride, 0.1 m sodium cacodylate pH 6.5, 2 m ammonium sulfate, 16 °C	5% tacsimate, pH 7.0, 0.1 m HEPES pH 7.0, 10% PEG MME 5000, 16 °C	5% tacsimate, pH 7.0, 0.1 m HEPES, pH 7.0, 10% PEG MME 5000, 16 °C	0.02 m magnesium chloride, 0.1 m HEPES, pH 7.5, 22%, PAA 5100, 16 °C	5% tacsimate, pH 7.0, 0.1 m HEPES, pH 7.0. 10% PEG MME, 16 °C	0.1 m succinic acid, pH 7.0, 15% PEG 3350, 16 °C
Cryo-protection solution	26% sucrose	25% glycerol	25% glycerol	15% glycerol	20% ethylene glycol	20% glycerol

*^a^* Values in parentheses correspond to the highest resolution shell.

*^b^ R*_merge_ = Σ*_hkl_*Σ*_i_*|*I_i_*(*hkl*) − 〈*I(hkl*)〉|/Σ*_hkl_*Σ*_i_*|〈*I_i_*(*hkl*)〉 , where *I_i_*(*hkl*) is the intensity for the *i*th measurement of an equivalent reflection with indices *h*, *k*, and *l*.

*^c^ R* = Σ*_hkl_*‖*F*_obs_| − |*F*_calc_‖/Σ*_hkl_*|*F*_obs_|, where *F*_obs_ and *F*_calc_ are observed and calculated structure factors, respectively. *R*_free_ is calculated analogously for the test reflections, which were randomly selected and excluded from the refinement.

*^d^* Ligands include all atoms, excluding protein and water atoms.

**TABLE 4:** **Crystallization conditions, data collection, and refinement statistics** ASU indicates asymmetric unit. BisTris is 2-[bis(2-hydroxyethyl)amino]-2-(hydroxymethyl)propane-1,3-diol; CAPS is 3-(cyclohexylamino)propanesulfonic acid.

	*Ba*IMPDHΔL ·IMP·7	*Clp*IMPDHΔL·IMP·1	*Clp*IMPDHΔL·IMP·2	*Cj*IMPDHΔS ·IMP·2	*Cj*IMPDHΔS ·IMP·4
**Data collection**					
Space group	*P*2	*P1*	*P*4_3_	*P*3_1_2	*I*422
Cell dimensions					
*a, b, c* (Å)	111.43, 56.23, 111.39	88.12, 89.25, 99.19	77.61, 77.61, 222.73	114.46, 114.46, 256.31	118.06, 118.06, 435.16
α, β, γ (°)	90.00, 89.83, 90.00	70.81, 72.66, 79.30	90.00, 90.00, 90.00	90.00, 90.00, 120.00	90.0, 90.0, 90.0
Protein molecules/ASU	4	8	4	4	3
Wavelength (Å)	0.9792	0.9792	0.9792	0.9793	0.9793
Resolution (Å)*^a^*	2.30 (2.30-2.34)	2.90 (2.90-2.95)	2.80 (2.80-2.85)	2.50 (2.50-2.54)	2.40 (2.40-2.44)
Unique reflections	59,534 (2462)	59,658 (2981)	32,619 (1635)	68,101 (3370)	58,784 (2723)
*R*_merge_*^b^*	0.118 (0.450)	0.132 (0.749)	0.104 (0.704)	0.109 (0.699)	0.146 (0.706)
〈*I/*σ*I*〉	7.5 (2.2)	7.6 (1.5)	9.4 (2.1)	10.2 (3.0)	7.3 (2.4)
Completeness (%)	95.7 (79.5)	98.5 (98.1)	99.8 (100.0)	99.9 (99.0)	96.7 (91.3)
Redundancy	3.1 (2.2)	2.0 (2.0)	3.4 (3.4)	8.8 (6.2)	9.1 (5.8)

**Refinement**					
Resolution (Å)	2.30 (2.30-2.33)	2.90 (2.89-2.93)	2.80 (2.79-2.87)	2.50 (2.50-2.53)	2.40 (2.40-2.44)
Reflections: work/test set	56,280/2997	59,543/3014	32,537/1652	64,427/3446	55,768/2968
*R*_work_/*R*_free_*^c^*	0.173 (0.225)	0.184 (0.243)	0.173 (0.223)	0.180 (0.218)	0172 (0.212)
No. of atoms: protein/ligands*^d^*/water	9723/272/299	20361/434/137	10037/208/81	10595/296/294	8026/199/202
Average *B* factor (Å^2^): protein/ligands/water	41.4/47.3/41.3	54.8/54.7/35.8	62.3/64.6/45.7	66.1/70.8/52.1	37.2/53.4/35.6
Bond lengths (Å)	0.008	0.003	0.003	0.008	0.008
Bond angles (°)	1.155	0.86	0.67	1.153	1.15
Most favored	96.4	96.05	97.03	96.2	97.1
Outliers	0.31	0.00	0.00	0.67	0.29
PDB code	4MY8	4Q33	4Q32	4MZ8	4MZ1
Crystallization conditions	0.1 m sodium/potassium phosphate, pH 6.2, 10% PEG 3,000, 16 °C	5% tacsimate, pH 7.0, 0.1 m HEPES, pH 7.0, 10% PEG MME 5000, 16 °C	0.1 m ammonium acetate, 0.1 m BisTris, pH 5.5, 17% PEG 10,000, 16 °C	1.6 m ammonium sulfate, 0.1 m MES, pH 6.5, 10% dioxane, 16 °C	0.2 m lithium sulfate, 0.1 m CAPS pH, 10.5, 1.2 m sodium/0.8 m potassium phosphate, 16 °C
Cryo-protection solution	25% glycerol	25% glycerol	20% glycerol	20% sucrose	20% sucrose

*^a^* Values in parentheses correspond to the highest resolution shell.

*^b^ R*_merge_ = Σ*_hkl_*Σ*_i_*|*I_i_*(*hkl*) − 〈*I(hkl*)〉|/Σ*_hkl_*Σ*_i_*|〈*I_i_*(*hkl*)〉 , where *I_i_*(*hkl*) is the intensity for the *i*th measurement of an equivalent reflection with indices *h*, *k*, and *l*.

*^c^ R* = Σ*_hkl_*‖*F*_obs_| − |*F*_calc_‖/Σ*_hkl_*|*F*_obs_|, where *F*_obs_ and *F*_calc_ are observed and calculated structure factors, respectively. *R*_free_ is calculated analogously for the test reflections, which were randomly selected and excluded from the refinement.

*^d^* Ligands include all atoms, excluding protein and water atoms.

**TABLE 5:** **Crystallization conditions, data collection, and refinement statistics for *Vc*IMPDHΔL complexes** ASU indicates asymmetric unit.

	*Vc*IMPDHΔL·IMP·NAD^+^	*Vc*IMPDHΔL·XMP·NADH
**Data collection**		
Space group	*P*42_1_2	*P*42_1_2
Cell dimensions		
*a, b, c* (Å)	121.33, 121.33, 94.47	91.26, 91.26, 171.21
α, β, γ (°)	90.00, 90.00, 90.00	90.00, 90.00, 90.00
Protein molecules/ASU	2	2
Wavelength (Å)	0.9792	0.9792
Resolution (Å)*^a^*	2.32 (2.32-2.36)	1.62 (1.65-1.62)
Unique reflections	31196 (1529)	92477 (4551)
*R*_merge_*^b^*	0.104 (0.918)	0.067 (0.743)
〈*I/*σ*I*〉	8.8 (2.1)	10.8 (2.8)
Completeness (%)	100 (100)	100 (100)
Redundancy	8.7 (6.6)	8.2 (7.8)

**Refinement**		
Resolution (Å)	2.32 (2.32-2.38)	1.62 (1.62-1.66)
Reflections: work/test set	29325/1553	87862/4552
*R*_work_/*R*_free_*^c^*	0.200 (0.270)	0.151 (0.181)
No. of atoms: protein/ligands*^d^*/water	5064/136/178	5208/165/563
Average *B* factor (Å^2^): protein/ligands/water	43.9/58.4/42.1	23.3/28.6/34.7
Bond lengths (Å)	0.016	0.015
Bond angles (°)	1.852	1.936
Most favored	96.8	97.6
Outliers	0.1	0.0
PDB code	4QNE	4X3Z
Crystallization conditions	0.77 m sodium/potassium phosphate, 0.15 m Tris-HCl, pH 8.0, 6% MPD, 16 °C	1.03 m sodium/potassium phosphate, pH 5.0, 0.15 m sodium malate, 3% PEG 300, 16 °C
Soak with 200 mm NAD^+^ solution	15 min, 20 °C	5 days, 16 °C
Cryo-protection solution	25% sucrose	25% glycerol

*^a^* Values in parentheses correspond to the highest resolution shell.

*^b^ R*_merge_ = Σ*_hkl_*Σ*_i_*|*I_i_*(*hkl*) − 〈*I(hkl*)〉|/Σ*_hkl_*Σ*_i_*|〈*I_i_*(*hkl*)〉 , where *I_i_*(*hkl*) is the intensity for the *i*th measurement of an equivalent reflection with indices *h*, *k*, and *l*.

*^c^ R* = Σ*_hkl_*‖*F*_obs_| − |*F*_calc_‖/Σ*_hkl_*|*F*_obs_|, where *F*_obs_ and *F*_calc_ are observed and calculated structure factors, respectively. *R*_free_ is calculated analogously for the test reflections, which were randomly selected and excluded from the refinement.

*^d^* Ligands include all atoms, excluding protein and water atoms.

##### Data Collection, Structure Solution, and Refinement

Prior to flash-cooling in liquid nitrogen, all crystals were cryoprotected in an appropriate solution (Tables 3–5). The crystals were mounted on Litholoops (Molecular Dimensions, Apopka, FL). All the x-ray diffraction experiments were performed at the Structural Biology Center ID-19 beamline at the Advanced Photon Source, Argonne National Laboratory (34). The HKL3000 suite (35) was used to process the diffraction images and to merge and scale intensities. Intensities were converted to structure factor amplitudes in the Truncate program from the CCP4 package (36). The crystal data statistics are given in Tables 3–5.

The structures were solved by molecular replacement (MR) using the HKL3000 program suite (35) from data collected from unlabeled protein crystals that diffracted up to 1.62–2.90 Å (Tables 3–5) using the structure of *Ba*IMPDH in complex with XMP (PDB code 3TSD) as a search model (with the exception of *Vc*IMPDHΔL·IMP·NAD^+^, for which the *Vc*IMPDH with IMP (PDB code 4IX2) was used as a model). The structures were manually adjusted to fit to electron density and to stereochemistry by using COOT (37) and refined by PHENIX (38) or REFMAC5.5 (39, 40) as a part of the CCP4 suite (36). During the refinement, the correctness of stereochemistry was examined with MolProbity (41). To reduce the model bias, all structures were subjected to 1–2 cycles of simulated annealing with the starting temperature of 5000 K at early stages of the refinement. The refinement statistics for the converged final models are given in Tables 3–5. The atomic coordinates and structure factors have been deposited in the Protein Data Bank (PDB) and the accession codes are listed in Tables 3–5.

##### Steady-state Enzyme Assays

Kinetic studies were performed in an assay buffer (50 mm Tris/HCl, pH 8.0, 100 mm KCl, 3 mm EDTA, 1 mm DTT) containing varied concentrations of IMP and NAD^+^ at 25 °C. NADH production was monitored by the increase in absorbance at 340 nm (ϵ = 6.22 mm^−1^ cm^−1^). Kinetic parameters were determined by collecting initial velocity data at varying concentrations of IMP (5–1000 μm) and NAD^+^ (100–5000 μm). Because IMPDH enzymes display substrate inhibition with respect to NAD^+^, the method described by Kerr *et al.* (42) was used to determine kinetic constants (10).

##### Determination of IC_50_

IC_50_ determinations were performed as described (11). Enzyme inhibition was assessed by monitoring the production of NADH by absorbance at varying inhibitor concentrations (50 pm to 5 μm). Each enzyme was incubated with an inhibitor for 10 min at room temperature prior to the addition of substrates. The following buffer conditions were used: 50 mm Tris/HCl, pH 8, 100 mm KCl, 1 mm DTT, 3 mm EDTA, 10 nm enzyme, 1 mm IMP. The NAD^+^ concentration was adjusted to ∼2.5 times of the value of *K_m_* for NAD^+^. IC_50_ values were calculated for each inhibitor according to Equation 1 using the SigmaPlot program (SPSS, Inc.),

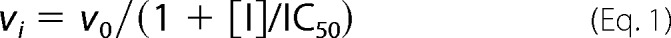
 where *v_i_* is the initial velocity in the presence of inhibitor [I] and *v*_0_ is the initial velocity in the absence of inhibitor.

## RESULTS

### 

#### 

##### Protein Selection and Design of CBS Deletion Variants

IMPDHs from four bacterial pathogens were selected to investigate the potential of *Cp*IMPDH-specific inhibitors as antibacterial agents. Three enzymes, *Ba*IMPDH, *Cj*IMPDH, and *Clp*IMPDH, possess the required IMSM (Ala-253/Tyr-445′, Ala-246/Tyr-440′, and Ala-248/Tyr-440′, respectively, analogous to Ala-165/Tyr-358′ in *Cp*IMPDH and therefore should be sensitive to the *Cp*IMPDH-specific inhibitors (4, 5, 11). *Vc*IMPDH contains Ser-252/Leu-446′ at these critical positions (Fig. 2) and therefore is resistant to these compounds and was used as a negative control.

Deletion of the CBS domain produced constructs showing improved properties and facilitated the crystallization of IMPDHs. Two deletion constructs, ΔL and ΔS, were prepared for each enzyme and cloned into the pMCSG7 vector as described under “Experimental Procedures” (33). In construct ΔL, the entire CBS domain (∼120 residues) was removed (Fig. 1*C*). Construct ΔS was designed to retain ∼20 residues at the C terminus of the second CBS domain that form a small, generally well ordered α-helix (α7; Fig. 1*D*), and the rest of the CBS domain (∼100 residues) was deleted. In both constructs, the deleted portions were replaced with an “SGG” connecting segment (Tables 1 and 2).

##### Expression and Purification

Soluble recombinant wild type and deletion mutant proteins were expressed and purified utilizing the high throughput pipeline developed at the Center for Structural Genomics of Infectious Diseases (33). Wild type enzymes *Ba*IMPDH, *Cj*IMPDH, and *Clp*IMPDH were purified as described previously (11). Both *Ba*IMPDHΔL and *Ba*IMPDHΔS forms were purified and crystallized. Only the ΔL deletion construct could be successfully purified and crystallized for *Clp*IMPDH and *Vc*IMPDH, and only the ΔS deletion mutant of *Cj*IMPDH exhibited satisfactory solubility and could be crystallized. All structures of protein·inhibitor complexes were obtained by co-crystallization with ∼10 times molar excess of IMP and ∼4 times molar excess of the inhibitor. The structures of the tertiary complexes of *Vc*IMPDHΔL with IMP/NAD^+^ and XMP/NAD^+^ were obtained by soaking crystals containing the *Vc*IMPDHΔL·IMP complex with a solution containing NAD^+^ for 15 min and 5 days, respectively. As observed previously, deletion of the CBS domain facilitated crystallization. Indeed, all 13 structures described here are either ΔL (9) or ΔS (3) mutants.

##### Kinetic Characterization of Bacterial IMPDHs and Their ΔCBS Variants

The kinetic properties of all four wild type IMPDHs are similar to each other as well as to those of other microbial enzymes (10). Deletion of the CBS domain does not significantly affect the steady-state kinetic parameters of the IMPDHs, with most parameters changed by less than a factor of 2 (Table 6), and only one parameter, the value of *K_ii_* for NAD^+^ for *Cj*IMPDHΔS, was changed by more than a factor of 3.

**TABLE 6:** **Kinetic parameters of bacterial IMPDHs and their ΔCBS constructs**

Enzyme	*k*_cat_	IMP, *K_m_*	NAD^+^, *K_m_*	NAD^+^ *K_ii_*
	*s*^−*1*^	μ*m*	μ*m*	*mm*
*Ba*IMPDH	5.3 ± 0.1	64 ± 16	550 ± 100	3.9 ± 0.8
*Ba*IMPDHΔS	6.1 ± 0.3	61 ± 4	560 ± 50	5.3 ± 0.7
*Ba*IMPDHΔL	4.5 ± 0.2	150 ± 20	460 ± 50	3.8 ± 0.5
*Cj*IMPDH	2.2 ± 0.1	55 ± 5	220 ± 30	20 ± 2
*Cj*IMPDHΔS	1.9 ± 0.1	27 ± 3	520 ± 70	6.4 ± 0.7
*Clp*IMPDH	2.9 ± 0.2	100 ± 10	370 ± 40	13 ± 2
*Clp*IMPDHΔL	1.8 ± 0.1	49 ± 4	510 ± 60	9 ± 1
*Vc*IMPDH*^a^*	2.1 ± 0.2	80 ± 10	1200 ± 200	ND*^b^* †
*Vc*IMPDHΔL	5.2 ± 0.3	87 ± 13	1100 ± 100	13 ± 3

*^a^* Data are from Ref. 11.

*^b^* ND means not determined.

##### Inhibition of Bacterial IMPDHs and Their ΔCBS Variants

We surveyed the inhibition of the wild type enzymes and their ΔCBS variants by seven *Cp*IMPDH inhibitors belonging to four chemical classes that originated from the high throughput screen against *Cp*IMPDH (Fig. 3 and Table 7) (24). A common feature of these inhibitors is a modular structure with two aromatic moieties connected by a linker. Inhibitor **1** is representative of an A-class scaffold with a 1,2,3-triazole linker (26). The remaining six compounds contain an amide linker. These include inhibitors from the C- and D-class with benzimidazole and phthalazinone moieties, **2** (5) and **3** (29), respectively, and a benzoxazole-based Q-class compound, **7** (28). The final three inhibitors, **4**, **5**, and **6** are representatives of a P-class with urea-based scaffold (Fig. 3) (30). It is important to point out that antimicrobial activity of a set of 140 *Cp*IMPDH inhibitors (including **1**-**7**) against *B. anthracis* Sterne 7702 was recently assessed (9). Several compounds, with **1** among them, had values of minimum inhibitory concentration less than or equal to 2 μm (9).

**TABLE 7:** **Inhibition of bacterial IMPDHs and their ΔCBS variants by *Cp*IMPDH-specific inhibitors** Assay conditions are described under “Experimental Procedures.”

Enzyme	IC_50_						
	1	2	3	4	5	6	7
	*nm*						
*Ba*IMPDH*^a^*	63 ± 12	67 ± 14	670 ± 80	19 ± 5	47 ± 5	20 ± 11	23 ± 4
*Ba*IMPDHΔS	137 ± 8	100 ± 4	800 ± 40	65 ± 11	40 ± 3	28 ± 4	38 ± 4
*Ba*IMPDHΔL	43 ± 3	72 ± 13	700 ± 100	ND*^b^*	10 ± 1	6 ± 1	27 ± 6
*Cj*IMPDH	140 ± 30	51 ± 9	169 ± 20	36 ± 8	22 ± 3	29 ± 4	13 ± 2
*Cj*IMPDHΔS	107 ± 8	42 ± 2	200 ± 20	67 ± 13	39 ± 3	46 ± 4	19 ± 2
*Clp*IMPDH	74 ± 33	570 ± 20	1700 ± 230	15 ± 1	34 ± 6	24 ± 10	54 ± 4
*Clp*IMPDHΔL	364 ± 20	450 ± 50	3400 ± 190	44 ± 8	92 ± 7	66 ± 3	95 ± 6
*Vc*IMPDH	>5000	>5000	>5000	>5000	>5000	>5000	>5000
*Vc*IMPDHΔL	>5000	>5000	>5000	>5000	>5000	>5000	>5000
*Cp*IMPDH*^c^*	9 ± 2	10 ± 1	24 ± 6	11 ± 5	5 ± 2	8 ± 2	6 ± 1

*^a^* Values are from Ref. 9.

*^b^* ND means not determined.

*^c^* Purification is as described in Ref. 31.

Enzyme inhibition was assessed by monitoring the production of NADH by absorbance at varying inhibitor concentrations. As expected, no inhibition was observed for either *Vc*IMPDH or *Vc*IMPDHΔL. *Ba*IMPDH, *Cj*IMPDH, and *Clp*IMPDH were all sensitive to the inhibitors, although the affinities were noticeably lower than observed for *Cp*IMPDH. Despite the sequence similarity of the inhibitor-binding sites, significant differences were observed in the structure-activity relationships of *Ba*IMPDH, *Cj*IMPDH, and *Clp*IMPDH. For *Ba*IMPDH variants, the IC_50_ values ranged from 6 to 800 nm, with **6** being the most potent and **3** the least potent inhibitor. Likewise, for the wild type and ΔL mutant of *Clp*IMPDH, **6** was the most potent, and **3** was the poorest inhibitor, although the IC_50_ values were higher for this enzyme (24–3400 nm). In the case of *Cj*IMPDH variants, the values of IC_50_ were between 13 nm for **7** and 200 nm for **3** (Table 7).

Compound **6** had the broadest spectrum, with the values of IC_50_ for all the three bacterial wild type enzymes ranging between 20 and 29 nm. Compound **7** was also a potent inhibitor of all three enzymes (IC_50_ ranging from 13 to 54 nm). In contrast, **2** displayed the most variability between bacterial wild type IMPDHs, with IC_50_ values between 51 and 570 nm. Compound **3** was the second most selective, with IC_50_ values between 169 and 1700 nm (Table 7). No significant differences were observed between the IC_50_ values for the WT and ΔS variants. Curiously, **5** and **6** displayed significantly lower (>3-fold) values of IC_50_ for *Ba*IMPDHΔL than *Ba*IMPDH, and the value of IC_50_ for **1** was higher in *Clp*IMPDHΔL than *Clp*IMPDH.

##### Overall Structure

The 13 crystal structures (resolution range 1.62–2.90 Å, Tables 3–5) reported here include the apo-form of *Ba*IMPDHΔS, the two *Vc*IMPDHΔL complexes with IMP/NAD^+^ and XMP/NAD^+^, and 10 IMP·inhibitor complexes (E·IMP·**I**), six *Ba*IMPDHΔL (**1**, **2**, **3**, **5**, **6**, and **7**), two *Cj*IMPDHΔS (**2** and **4**), and two *Clp*IMPDHΔL (**1** and **2**; Tables 3–5). The SGG motif, which was engineered to replace the deleted CBS domain, is well ordered and visible in all the structures of ΔL mutants, but it has varying degrees of disorder in the structures of ΔS variants. The apo *Ba*IMPDHΔS structure is the most flexible, with significant differences in all four subunits in the asymmetric unit (r.m.s.d. for Cα atoms from 0.99 to 1.08 Å). The apo structure also contains more disordered regions than the liganded structures.

**FIGURE 4.**
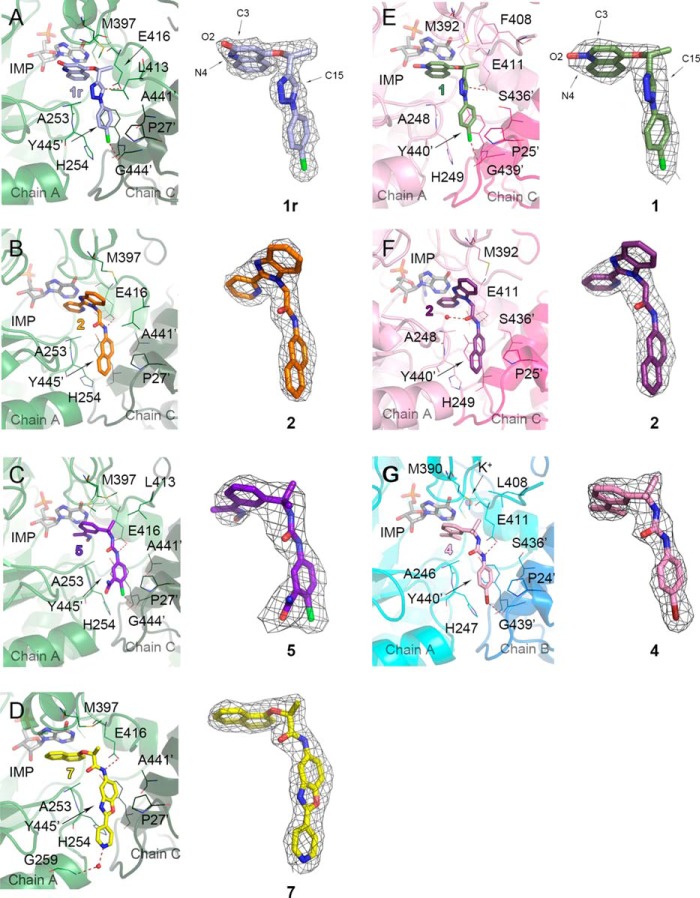
**Binding of inhibitors in bacterial IMPDHs.** *A, Ba*IMPDHΔL·IMP·**1r**, where **1r** (*light blue*) indicates a radiation-modified **1**. Atoms involved in the *N*-oxide rearrangement (O2, C3, and N4) and C15 are labeled. *B, Ba*IMPDHΔL·IMP·**2** (*orange*). *C, Ba*IMPDHΔL·IMP·**5** (*purple-blue*). *D, Ba*IMPDHΔL·IMP·**7** (*yellow*). *E, Clp*IMPDHΔL·IMP·**1** (*olive*), atoms O2, C3, N4, and C15 are labeled. *F, Clp*IMPDHΔL·IMP·**2** (*purple*). *G, Cj*IMPDHΔS·IMP·**4** (*pink*). Protein chains are in a cartoon representation. Adjacent monomers forming the active site are shown in different colors (*green* and *dark green* for *Ba*IMPDHΔL, *light pink* and *dark pink* for *Clp*IMPDHΔL, and *teal* and *marine blue* for *Cj*IMPDHΔS) and labeled. Ligand molecules are shown in a *stick* representation. Hydrogen and halogen bonds are depicted as *red dashed lines*. A *prime* denotes a residue from the adjacent monomer. Water molecules in *D* and *F* and a potassium ion in *G* are shown as *red* and *purple* spheres, respectively. For each panel, 2m*F_o_* − D*F_c_* electron density map contoured at the 1 σ level for each inhibitor is shown on the *right*.

**FIGURE 5.**
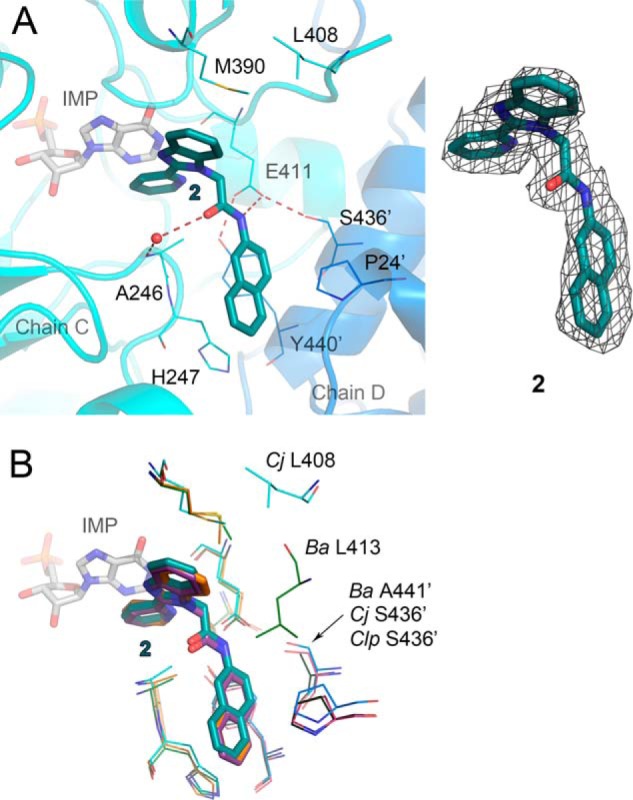
**Interactions of inhibitor 2 with three bacterial proteins.** *A, Cj*IMPDHΔS·IMP·**2** complex. Chains C (*teal*) and D (*marine blue*) are shown in a cartoon representation. Molecules of IMP (*gray*) and **2** (*dark teal*) are shown as *sticks*. Residues involved in inhibitor binding are shown as *lines*. Water molecule is depicted as a *red sphere*. Hydrogen bonds are represented as *red dashed lines*. A *prime* denotes a residue from the adjacent monomer. 2m*F_o_* − D*F_c_* electron density map contoured at the 1 σ level for **2** is shown on the *right. B,* overlay of three bacterial structures with **2**. Only variable residues are labeled. Color designations are as follows: *Cj*IMPDHΔS·IMP·**2** as in *A*; *Ba*IMPDHΔL·IMP·**2**, chain A, *green*; chain C, *dark green*; **2**, *orange*; *Clp*IMPDHΔL·IMP·**2**, chain A, *pink*; chain C, *dark pink*; **2**, *purple*.

**FIGURE 6.**
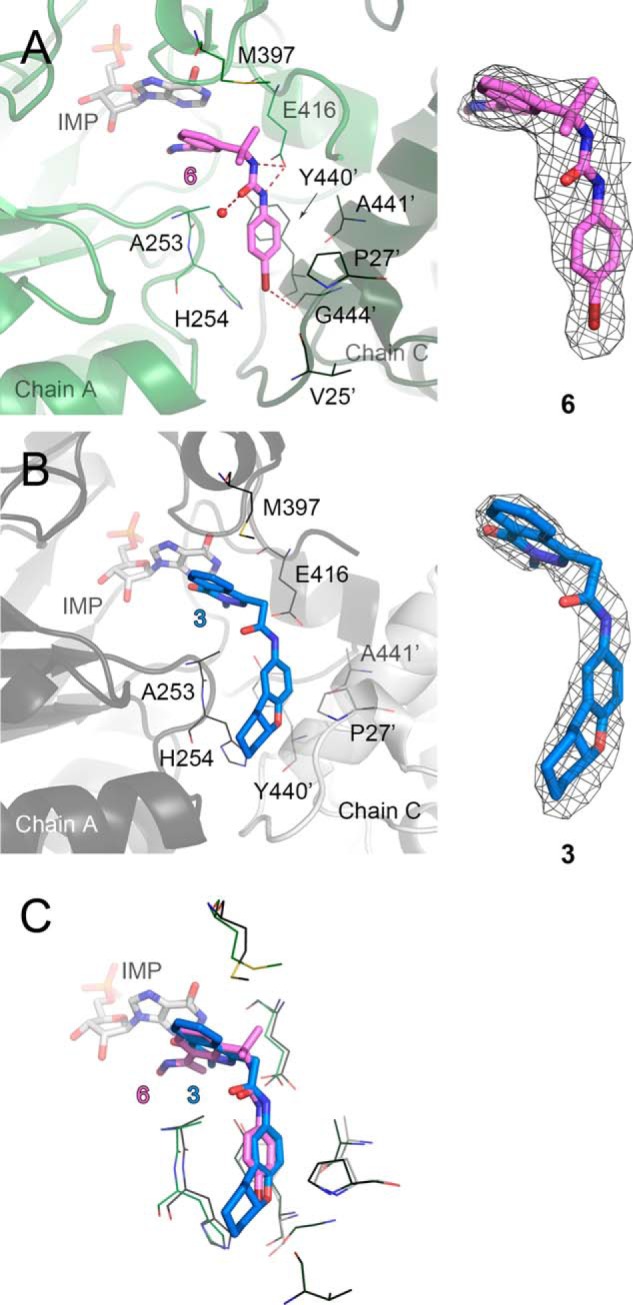
**Binding of the most and the least potent inhibitors in *Ba*IMPDHΔL.** *A, Ba*IMPDHΔL·IMP·**6** complex. Chains A (*green*) and C (*dark green*) are shown in a cartoon representation. *B, Ba*IMPDHΔL·IMP·**3** complex. Chains A (*dark gray*) and C (*light gray*) are shown in a cartoon representation. *C,* overlay of the two structures. Only the residues participating in binding of the inhibitors are shown. Color code as in *A* and *B*. Molecules of IMP (*light gray*), **6** (*violet*), and **3** (*aquamarine*) are shown as *sticks*. Residues involved in inhibitor binding are represented as *lines*. A *prime* denotes a residue from the adjacent monomer. Hydrogen and halogen bonds are depicted as *red dashed lines*. Water molecule is represented as a *red sphere. A* and *B*, 2m*F_o_* − D*F_c_* electron density map contoured at the 1 σ level for each inhibitor is shown on the *right*.

**FIGURE 7.**
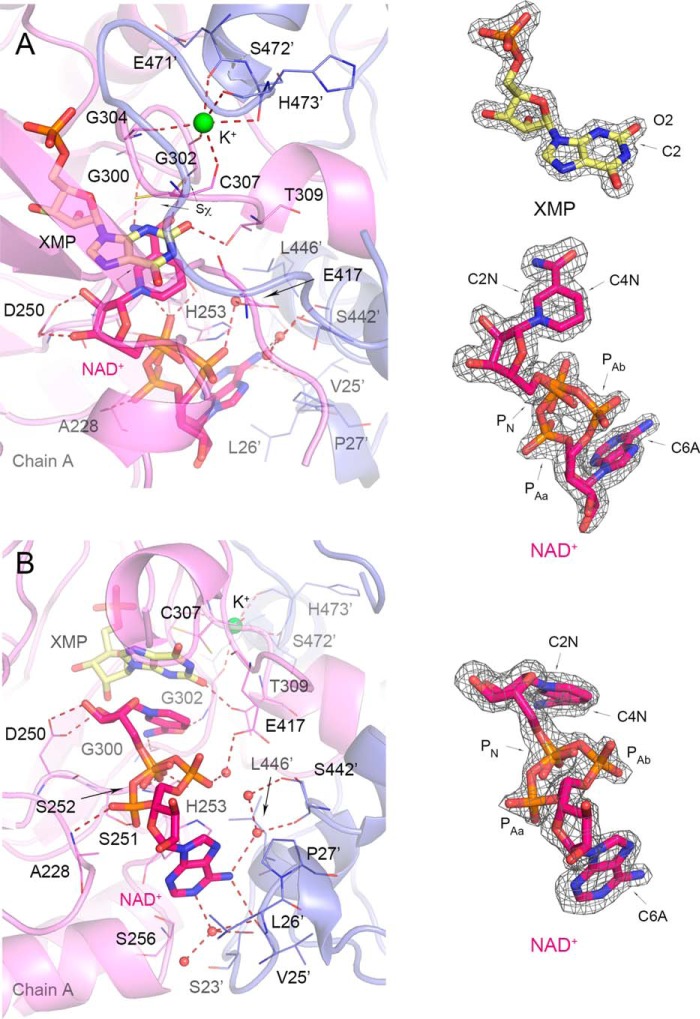
**Cofactor binding in *Vc*IMPDHΔL·XMP·NAD^+^.** *A,* top view of the active site with XMP product and K^+^ site visible. Chain A (*violet*) and symmetry-generated adjacent chain (*slate blue*) are shown in a cartoon representation. Residues are represented as *lines*. A *prime* denotes a residue from the adjacent monomer. XMP (*light yellow*) and NAD^+^ (*magenta*) are shown as *sticks*. Water molecules and K^+^ ion are shown as *red* and *lime spheres*, respectively. Hydrogen bonds and K^+^ coordinating bonds are depicted as *red dashed lines. B,* side view of the active site detailing NAD^+^ binding. Color code and depiction as in *A. A*, 2m*F_o_* − D*F_c_* electron density map contoured at the 2 σ level for XMP (*yellow*) is shown on the *right*. Also shown on the *right* for both panels is 2m*F_o_* − D*F_c_* electron density map contoured at the 1.5 σ level for NAD^+^ in top (*A*) and side view (*B*). Atoms discussed in text are labeled.

**FIGURE 8.**
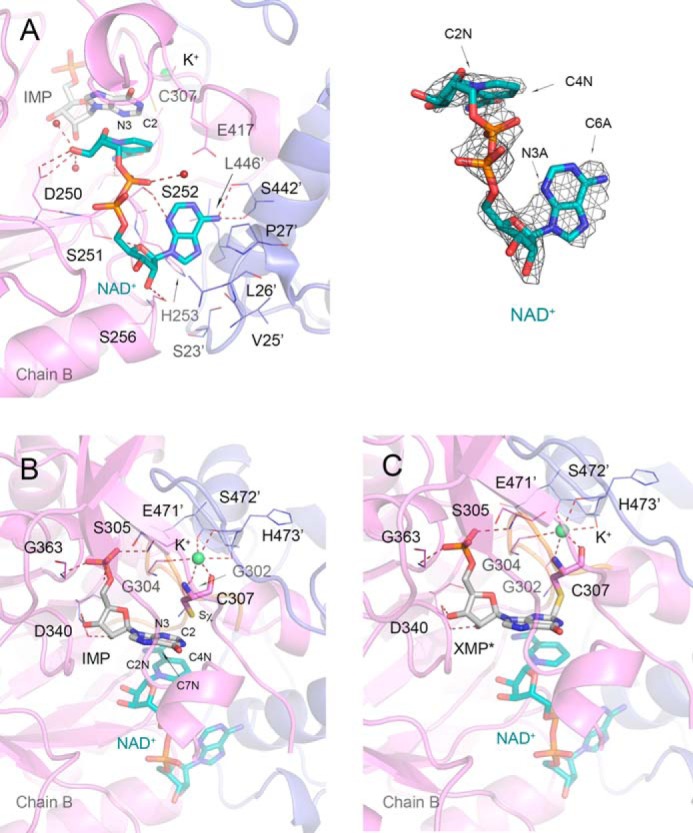
**NAD^+^ binding and two orientations of the active site cysteine 307 in *Vc*IMPDHΔL·IMP·NAD^+^.** *A,* NAD^+^ site in *Vc*IMPDHΔL·IMP·NAD^+^. Chain B (*violet*) and symmetry-generated adjacent chain (*slate blue*) are shown in a cartoon representation. Residues are represented as *lines*. A *prime* denotes a residue from the adjacent monomer. IMP (*gray*) and NAD^+^ (*teal*) are shown as *sticks*. Water molecules and K^+^ ion are shown as *red* and *lime spheres*, respectively. Hydrogen bonds and K^+^ coordinating bonds are depicted as *red dashed lines*. On the *right* 2m*F_o_* − D*F_c_* electron density map contoured at the 1 σ level for the NAD^+^ molecule is shown. *B,* IMP (*gray*) and free Cys-307 modeled at 60% occupancy. For clarity, NAD^+^ (*teal*) is shown at 50% transparency. *C,* covalent E-XMP* intermediate (*gray*) formed between the Sχ atom of Cys-307 and the C2 atom of IMP modeled at 40% occupancy. NAD^+^ is shown as in *B*. Protein chains, residues, and K^+^ ion are represented as in *A*. Active site loop containing catalytic Cys-307 is shown in *orange.* Ligand molecules and Cys-307 residue are shown in a *stick* representation. Atoms discussed in text are labeled.

The active site is ordered in all structures of complexes, except for 12–15 residues of the active site flap. The electron densities for the ligand and active site residues are well defined (Figs. 4–8). IMP binds in an essentially identical mode to that observed previously for *Ba*IMPDH and therefore will not be discussed further (11). The residues involved in IMP binding are conserved in all IMPDHs, and analogous interactions are also observed in eukaryotic IMPDHs. The only difference is that Glu-416 (*Ba*IMPDH numbering) is replaced by glutamine in eukaryotic IMPDHs. As discussed below, this residue may contribute to inhibitor selectivity.

##### Inhibitor Binding

Generally, the IMPDH inhibitor complexes have very similar structures, with r.m.s.d for Cα atoms of the A chains ranging from 0.25 to 1.32 Å. These similarities most likely derive from the high sequence identity between these IMPDHs (62% for the *Ba*IMPDH and *Cj*IMPDH, 67% for *Ba*IMPDH and *Clp*IMPDH, and 56% for *Ba*IMPDH and *Vc*IMPDH) and the comparable structural states. Each active site of the complex contains IMP and an inhibitor. In each case, the inhibitor interacts with the hypoxanthine ring of IMP via one aromatic group, and the other aromatic group interacts with the IMSM tyrosine, as observed previously in *Cp*IMPDH (28, 31). The linker (amide or triazole) is adjacent to the IMSM alanine residue, as also found in *Cp*IMPDH. The linker interacts with the bacterially conserved glutamate residue (Glu-416 in *Ba*IMPDH) in all of inhibitor complexes. The specific role of this residue in the active site is not well understood. It is important to note that the inhibitors do not mimic the cofactor-binding mode observed in eukaryotic enzymes, which is mainly localized within one monomer (see below), but instead it binds to both subunits. The structurally distinct binding pocket has been consistently observed in the bacterium-like *Cp*IMPDH (28, 31) and now in three bacterial IMPDHs presented here, yet the functional association of different parts of this binding site has remained unexplained until now.

##### Interaction of Benzimidazole-based Compound **2** with Three Bacterial IMPDHs

As noted above, the potency of inhibitor **2** varies by an order of magnitude among the three bacterial enzymes containing the IMSM motif (Table 7). *Cj*IMPDHΔS has the highest affinity for **2**, followed by *Ba*IMPDHΔL and *Clp*IMPDHΔS (IC_50_ = 42, 72, and 450 nm, respectively). The structures of compound **2** in complex with *Ba*IMPDHΔL, *Cj*IMPDHΔS, and *Clp*IMPDHΔL were determined to reveal differences in the enzyme-inhibitor interactions that may account for the different potencies.

The overall binding mode of **2** is very similar, and the residues making direct contact with the inhibitor are conserved among the three enzymes (Figs. 4, *B* and *F,* and 5). The 2-pyridinyl substituent of **2** interacts with the hypoxanthine ring of IMP in a nonclassical off-centered face-to-face orientation in all three complexes, and the benzimidazolyl moiety contacts the side chain of Met-397 (*Ba*IMPDH numbering). The naphthalenyl group binds similarly in all three bacterial IMPDHs (Figs. 4 and 5). One ring of the naphthalenyl moiety of **2** makes π/π-type contacts with Tyr-445′ in an orientation that is midway between the face-to-face and edge-to-face geometries, whereas the other ring is sandwiched between His-254 and Pro-27′.

Subtle differences are observed within each subunit of the asymmetric unit of a given enzyme as well as in the protein-inhibitor interactions between enzymes. For example, the amide oxygen of **2** forms a water-mediated hydrogen bond with the main chain nitrogen of Ala-253 in subunits E and G of *Ba*IMPDHΔL. This interaction is also present in the *Cj*IMPDHΔS complex in chains A, B, and C. However, this water-mediated hydrogen bond is only found in chain A in *Clp*IMPDHΔL, and the interaction is longer (Wat/O-Ala-248/N distance of 3.46 Å compared with 3.20 Å in chain A of *Ba*IMPDHΔL).

The compound **2** linker amide NH forms a hydrogen bond with one of the oxygen atoms of Glu-416 in all three enzymes (Glu-411 in *Cj*IMPDH and *Clp*IMPDH), but the distance does not directly correlate with affinity as follows: 2.75 Å in *Ba*IMPDHΔL; 2.92 Å in *Cj*IMPDHΔS; and 3.20 Å in *Clp*IMPDHΔL. In *Cj*IMPDHΔS and *Clp*IMPDHΔL, this glutamate also forms a hydrogen bond with a serine residue from the adjacent monomer (Ser-436′ in both enzymes). This residue is replaced by Ala-441 in *Ba*IMPDHΔL, which may explain the shorter hydrogen bond to the inhibitor (Fig. 5*B*). The active site flap also displays different conformations that may influence inhibitor affinity. The flap (residues 401–412) is disordered in all eight subunits of the asymmetric unit of *Ba*IMPDHΔL. The first visible residue, Leu-413, is positioned above the amide linker on the opposite face of the inhibitor from Ala-253, which suggests that the disordered portion of the flap may transiently interact with the inhibitor (Fig. 5*B*). The flap may have a somewhat different interaction with **2** in *Cj*IMPDHΔS. The analogous residue, Leu-408, is visible in all four subunits (A–D). In subunits A and B, Leu-408 is situated above the linker amide as seen in *Ba*IMPDHΔL. However, Leu-408 is shifted away from the inhibitor in subunits C and D (C/D-averaged **2**/O-Leu-408/CD1 distance of 8.83 Å) (Fig. 5*A*). In *Clp*IMPDHΔL, the corresponding residue is Phe-408, which is visible only in subunits C and D. In subunit C, Phe-408 is positioned in the proximity of the **2** amide (**2**/O-Phe-408/centroid distance of 4.9 Å), although in chain D this residue faces away from the binding pocket (centroid distance of 11.2 Å). These structures confirm high conformational flexibility of the active site flap and suggest that the flap dynamics may also contribute to the differences in inhibitor affinities. Comparisons of inhibitor-binding modes and the flap conformations in different molecules in the crystal asymmetric unit indicate the dynamic nature of enzyme-ligand interactions. Some of these states may correspond to different steps in ligand binding or various ways the ligand can be accommodated in the active site.

##### Interaction of Bacterial IMPDHs with Other Inhibitors

To further explore the structural basis of inhibitor potency (Table 7), *Ba*IMPDHΔL was also co-crystallized with IMP and five other inhibitors, **1**, **3**, **5**, **6,** and **7**. In addition, the structures of *Clp*IMPDHΔL·IMP·**1** and *Cj*IMPDHΔS·IMP·**4** were also determined. Most of the interactions are similar to those observed in the complexes of **2**, so the following description will focus on the major differences.

##### Complexes with 1,2,3-Triazole-containing Inhibitor **1**

Compound **1** is a moderately potent inhibitor of *Ba*IMPDH variants (Table 7). Close examination of the electron density of the inhibitor in the *Ba*IMPDHΔL·IMP·**1** complex revealed that the *N*-oxide oxygen (O2) migrated from N4 to C3 (labeled **1r** in Fig. 4*A*). This rearrangement appears to have been caused by the x-ray radiation during data collection, because the compound used for crystallization was undoubtedly the *N*-oxide form of **1**. Similar radiation-induced *N*-oxide rearrangements have been observed in other systems (43, 44). The *N*-oxide rearrangement is not detected in the *Clp*IMPDHΔL·IMP·**1** complex, but this may be due to the lower resolution of this structure (Fig. 4*E*). Unlike the other inhibitors, **1** contains a triazole linker. Like an amide, the triazole has a strong dipole, which places a partial positive charge on C15–H. This carbon atom is on average 3.26 ± 0.11 Å from Glu-416 OE1 (*Ba*IMPDH numbering). A new interaction is observed between the 4-chloro substituent of the phenyl ring and the carbonyl group of Gly-444′ from the adjacent subunit (Fig. 4). The halogen substituent also interacts with the ring of His-254. The same interactions are present in the structure of *Clp*IMPDHΔL·IMP·**1**, although all the contacts are generally longer, consistent with the lower affinity of **1** for *Clp*IMPDH (Table 7).

##### Comparison of Structures with Three P-class Urea-derived Inhibitors **4, 5, 6**

The P-series includes the largest number of low nanomolar inhibitors of *Cp*IMPDH, although on average these compounds have 4-fold less affinity for *Ba*IMPDH than for *Cp*IMPDH (9). We have determined structures of the E·IMP·**I** complexes of *Ba*IMPDHΔL with **5** and **6** (Figs. 4*C* and 6*A*) and *Cj*IMPDHΔS with **4** (Fig. 4*G*). In all cases, both nitrogen atoms of the urea group can form hydrogen bonds with Glu-416 (Glu-411 in *Cj*IMPDH) and the IMP hypoxanthine ring interacts with the *sp*^2^ center and the aromatic centroid of the left-side ring (Figs. 3, 4, *C* and *G*, and 6, *A* and *C*). These interactions may account for the potency of the P-series. As observed in **1**, halogen bonds form with the substituents of the right-side rings (Fig. 3) and the carbonyl oxygen of Gly-444′ and C–*X*/π interactions are observed with the imidazole of His-254. Interestingly, the carboxamide group of **5** (Fig. 4*C*) does not appear to participate in any hydrogen bonds. This position is a promising site for further optimization of potency and pharmacological properties.

##### BaIMPDHΔL Complex with Phthalazinone-based Inhibitor **3**

Compound **3** is the least potent inhibitor of *Ba*IMPDHΔL, and there are several features of the E·IMP·**3** complex that may account for its low affinity. Instead of the π/π interactions observed for the other inhibitors, only the *N*-methyl and the carbonyl groups of the dihydrophthalazine portion of **3** contact the hypoxanthine ring of IMP. This interaction is a combination of *sp*^2^/π and *sp*^3^/π contacts and thus involves a smaller portion of the inhibitor than in **2** and **1**. The distances between the compound **3** amide linker and the side chain of Glu-416 are longer (2.84 Å in chain C but >3.40 Å in chains A, B, and D) than in other amide-containing complexes indicating possibly a smaller contribution from the hydrogen bonding interaction in the complex with **3**. Finally, the tetrahydrobenzyl ring of **3** interacts with His-254, Pro-27′, and Tyr-445′, but it does not appear to make any additional contacts with the protein (Fig. 6, *B* and *C*). These observations may explain the relatively low potency of **3**.

##### BaIMPDH Complex with a Benzoxazole Derivative, Compound **7**

Compound **7** is a potent inhibitor of *Ba*IMPDH, *Cj*IMPDH, and *Cp*IMPDH. The general binding mode of **7** to *Ba*IMPDHΔL is very similar to that of **2**. However, where Met-397 interacts with the benzimidazolyl moiety of **2**, it contacts only the (*S*)-methyl group of the amide linker in **7** (Fig. 4*D*). This interaction explains the preference for the *S* isomer of **7** (9, 28). Another notable difference between the complexes of **7** and **2** is that the 4-pyridyl substituent forms a water-mediated hydrogen bond with the main chain nitrogen atom of Gly-259, as observed in the complex with *Cp*IMPDH (Figs. 4*D* and 8*D*) (28).

##### Binding of NAD^+^ to VcIMPDHΔL

Despite the fact that *Vc*IMPDH does not possess the IMSM and does not bind *Cp*IMPDH inhibitors, the structures of this protein provide crucial information about the distinct inhibitor binding mode employed by bacterial enzymes. We obtained the first two crystal structures of a cofactor complex of a bacterial IMPDH by soaking crystals of *Vc*IMPDHΔL·IMP with 200 mm NAD^+^ for different lengths of time (15 min and 5 days). The longer incubation was done to improve structure resolution and electron density for the NAD^+^ molecule. The two *Vc*IMPDHΔL complexes with IMP/NAD^+^ and XMP/NAD^+^ were determined at 2.32 and 1.62 Å resolution, respectively. Each structure contains two protein chains (A and B) per asymmetric unit.

The high resolution *Vc*IMPDHΔL·XMP·NAD^+^ structure provides a detailed view of the active site and NAD^+^ conformation. Examination of the high quality electron density maps shows that XMP instead of IMP is present in the active site. This indicates that all the IMP substrate has been converted to the final product (XMP). This also means that both the dehydrogenase (hydride transfer) and the hydrolase reaction (hydrolysis of the E-XMP* intermediate) have occurred in the crystal. However, although NADH must have been produced concomitantly with XMP, the cofactor present in the structure is most likely NAD^+^ due to the large excess of this form used for soaking (200 mm NAD^+^ *versus* 4 mm IMP). XMP is essentially in the same orientation and makes contacts with the same residues as IMP in the structures of inhibitor complexes. However, there is an additional hydrogen bonding interaction present between the O2 of XMP and the side chain of Thr-309 (*Vc*IMPDH numbering) (Fig. 7*A*). The side chain of catalytic Cys-307 is in two conformations. In both conformations the Cys-307's Sχ atom faces away from the C2 carbon of XMP to avoid clash with the newly formed C2–O2 bond (average Sχ-C2 distance 3.52 ± 0.07 Å) (Fig. 7, *A* and *B*).

The structure contains a K^+^ ion, which is required for IMPDH activity (10, 45). The ion is bound between two subunits and is coordinated by several carbonyls, including one from the catalytic Cys-307 that helps position this residue for catalysis (Fig. 7, *A* and *B*). This location is very similar to that observed in previously reported structures (for example, see the structure of the E-XMP*·mycophenolic acid complex of Chinese hamster IMPDH, PDB code 1JR1 (46)).

**FIGURE 9.**
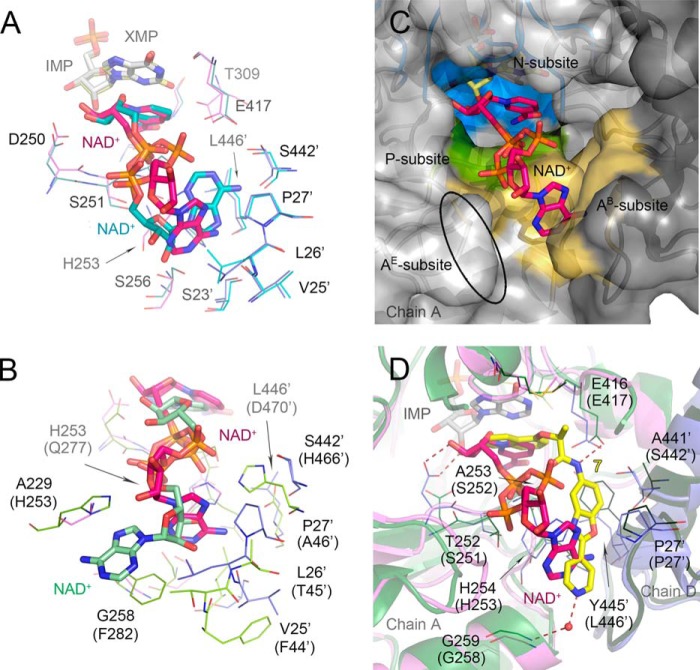
**Different mode of NAD^+^ binding in bacterial IMPDHs.** *A,* overlay of the cofactor position in *Vc*IMPDHΔL·XMP·NAD^+^ and *Vc*IMPDHΔL·IMP·NAD^+^. Only ligands (depicted as *sticks*) and the interacting residues (represented as *lines*) are shown. Color code is as follows: for XMP/NAD^+^ structure as in Fig. 7; for IMP/NAD^+^ structure, chain B (*teal*), symmetry-generated adjacent chain (*light blue*), NAD^+^ (*teal*), IMP (*gray*). Water molecules and hydrogen bonds are omitted for clarity. *B,* overlay of the cofactor position in *Vc*IMPDHΔL·IMP·NAD^+^ and hIMPDH2·CPR·NAD^+^ (PDB code 1NFB, as in Fig. 1*B*). Only the cofactors (depicted as *sticks*) and the interacting residues (represented as *lines*) are shown. Variable residues are labeled according to *Vc*IMPDH numbering with hIMPDH2 numbering in *parentheses*. IMP and CPR are omitted for clarity. Color code is as follows: for *Vc*IMPDHΔL as Fig. 7; for hIMPDH2, chain A (*green*), symmetry-generated adjacent chain (*lime*), NAD^+^ (*pale green*). *C,* migration of the cofactor A-subsite to the adjacent monomer. Chains A (*light gray*) and symmetry-generated adjacent chain (*dark gray*) are shown in a surface representation. XMP (*light yellow*) and NAD^+^ (*magenta*) are shown as *sticks*. Three NAD^+^ subsites N- (*blue*), P- (*green*), and the bacterial A^B^-subsite (*orange*) are represented. The *ellipsoid* depicts the localization of the eukaryotic A^E^-subsite. *D,* overlay of the cofactor and inhibitor positions in *Vc*IMPDHΔL·XMP·NAD^+^ and *B*aIMPDHΔL·IMP·**7**. Color code for the cofactor structure as in Fig. 7. Residues (shown as *lines*) are labeled according to *Ba*IMPDH numbering with *Vc*IMPDH numbering in *parentheses*. Chains A (*green*) and D (*dark green*) of *Ba*IMPDHΔL·IMP·**7** are shown in a cartoon representation. IMP (*light gray*), NAD^+^ (*magenta*), **7** (*yellow*) are shown as *sticks*. Hydrogen bonds are depicted as *red dashed lines*. Water molecule for the inhibitor is shown as a *red sphere*. Waters molecules for the cofactor structure are omitted for clarity.

Most importantly, this structure reveals a new NAD^+^ conformation and an A-subsite (Figs. 1*B* and 9) that are dramatically different from those reported previously for eukaryotic IMPDHs (PBD codes 1MEW (19) and 1NFB). The cofactor assumes a more compact conformation than usually found in dehydrogenases. The adenine ring in an anti-orientation and the cofactor adopt a semi-folded conformation (48) with the C6A–C2N distance of 11.41 ± 0.11 Å (Fig. 7). The corresponding distances in human type 2 (hIMPDH2) and *Tritrichomonas foetus* (*Tf*IMPDH) enzymes are considerably longer at 14.55 and 15.97 Å, respectively (PDB code 1NFB and 1MEW (19), respectively). This is a rare conformation for NAD^+^ and other adenine-containing nucleotides (48–50). Interestingly, a similar conformation of the cofactor was observed in the structure of the related enzyme, GMP reductase (51). Specific interactions between *Vc*IMPDHΔL and the nicotinamide, pyrophosphate, and adenosine portions of NAD^+^ will be described below.

Despite this unusual cofactor conformation, the interactions of the nicotinamide moiety with the N-subsite are essentially the same as those reported previously for eukaryotic IMPDHs, the nicotinamide stacks against the xanthine ring of XMP. The carboxamide moiety makes two hydrogen bonds to the main chain amido and carbonyl groups of conserved Gly-300 and Gly-302, respectively. The nicotinamide ribose is anchored through hydrogen bonds via its hydroxyl groups and Asp-250 (Fig. 7, *A* and *B*). The distance between the C4N of nicotinamide and the C2 of xanthine between which the hydride transfer step has occurred is 3.33 ± 0.01 Å.

The interactions of the P-subsite are also similar to previously reported cofactor structures, although two conformations are observed for the pyrophosphate moiety. Specifically, although the position of the phosphate group next to the nicotinamide (P_N_) stays the same, the phosphate group next to the adenosine (P_A_) exists in two orientations. In one orientation, referred to as P_Aa_ in Fig. 7, the phosphate moiety forms a hydrogen bond to the amido group of Ala-228 (chain A only), the carbonyl of Asp-250 (chain B only), and the side chain of Ser-251 (chains A and B). In the other orientation (P_Ab_ in Fig. 7), it shifts toward the interface between the monomers and makes water-mediated hydrogen bonds with the bacterially conserved Glu-417 (Glu-416 in *Ba*IMPDH). In both orientations the P_N_ group of NAD^+^ contacts the hydroxyl and the amido group of Ser-252. The corresponding interaction is also found in the structures of both hIMPDH2 and *Tf*IMPDH (PDB code 1NFB and 1MEW (19), respectively). However, this position corresponds to the IMSM alanine residue in *Cp*IMPDH and other bacterial IMPDHs.

The most striking difference is observed in the position of the cofactor adenosine moiety. In the eukaryotic IMPDHs, the A-subsite is located in the same monomer as the N-subsite, and the adenosine is stacked between residues capable of π/π or π/cation interactions (His-253/Phe-282 in hIMPDH2 and Arg-241/Trp-269 in *Tf*IMPDH (Figs. 1*B* and 9*B*) (19)). In contrast, the position of the A-subsite in *Vc*IMPDHΔL·XMP·NAD^+^ has migrated to a site in an adjacent monomer (Figs. 7 and 9, *A–C*). The adenine moiety makes two direct hydrogen bonds with the main chain carbonyl of Val-25′ (chain A) and the side chain of Ser-256 (chain B). There are also several water-mediated contacts involving the main chain carbonyls and the side chains of Ser-23′and Ser-442′ in both subunits. In addition, one side of the adenine ring participates in van der Waals interactions with the side chains of Leu-26′ and Pro-27′, whereas the other side π/π stacks with the side chain of His-253 in a face-to-face orientation (Fig. 7). (Note: His-253 does not refer to a histidine residue of the same number listed above for hIMPDH2; His-253 in *Vc*IMPDH corresponds to Gln-277 in hIMPDH2, whereas His-253 in hIMPDH2 corresponds to Ala-229 in *Vc*IMPDH.) Interestingly, ribose hydroxyl groups are exposed to the solvent and do not seem to make any direct contacts with the protein.

The second crystal structure (*Vc*IMPDHΔL·IMP·NAD^+^) was obtained after soaking crystals of *Vc*IMPDHΔL·IMP with NAD^+^ for a short period of time (15 min). Although this structure is a lower resolution (2.32 Å), it still provides important clues about the reaction and cofactor binding (Fig. 8). Careful inspection of electron density maps in this structure revealed that the average distance between the C2 of the hypoxanthine ring and Sχ of the catalytic Cys-307 is 2.71 ± 0.24 Å, which is significantly shorter than the same distance in the *Vc*IMPDHΔL·XMP·NAD^+^. It is also shorter than the average distance of 3.29 ± 0.08 Å observed in the structures of the IMP·inhibitor complexes but longer than a single C–S bond (1.83 Å) (47). This intermediate distance suggests that a mixture of a substrate and the covalent intermediate E-XMP* is observed, also indicating that the reaction in the crystal was captured at the dehydrogenase step. Like in the XMP/NAD^+^ structure, two conformations are observed for Cys-307, one of which is consistent with an E-XMP* covalent bond (Fig. 8, *B* and *C*). The density can be modeled with a 60–40% mixture of IMP and E-XMP*. This structure also contains a K^+^ ion that is coordinated in the same way as described earlier (Fig. 8, *B* and *C*).

The bound NAD^+^ cofactor assumes a similarly folded conformation as in the structure with XMP/NAD^+^. However, the adenine ring is flipped ∼180° and is now in a *syn* conformation. The C6A-C2N distance is shorter (9.42 ± 0.10 Å), indicating that the cofactor is in a more compact orientation (Fig. 8*A*). As a result, the adenosine moiety is found deeper in the A-subsite.

The position of the nicotinamide in *Vc*IMPDHΔL·IMP·NAD^+^is superimposable with the position of the same moiety in *Vc*IMPDHΔL·XMP·NAD^+^, and the same protein-ligand contacts are observed. The preservation of these interactions is expected given the conservation of the N-subsite and geometrical requirements for a hydride transfer. This structure clearly shows that the nicotinamide ring helps to position the hypoxanthine ring against Cys-307. In addition, the amide group may interact with N3 of the hypoxanthine ring (C7N-N3 distance of 3.48 ± 0.11 Å) to possibly activate C2, although this interaction has not been directly observed (Fig. 8*A*).

Only one conformation of the pyrophosphate group is observed in *Vc*IMPDHΔL·IMP·NAD^+^. The P_N_ group makes identical contacts with Ser-252 as found in the other *Vc*IMPDHΔL structure, although the P_A_ group exists in the P_Aa_-like orientation, although no interactions with either Ala-228, Asp-250, or Ser-252 are present.

Even though the adenosine in *Vc*IMPDHΔL·IMP·NAD^+^ is not positioned in exactly the same way as in the structure with XMP/NAD^+^, this moiety makes contact with the same set of residues as in *Vc*IMPDHΔL·XMP·NAD^+^. Specifically, the adenine amino group forms hydrogen bonds with both the side chain hydroxyl and the main chain carbonyl oxygen of Ser-442′, whereas the adenine's N3A atom contacts the side chain of Ser-252 (Fig. 8*A*). Hydrophobic contacts are also maintained and include interactions with Leu-26′, Pro-27′, and His-253. However, in this case His-253 rotates away and participates in an edge-to-face type of contact with the adenine ring. Because the adenosine moiety is positioned deeper into the binding pocket, the adenine ring is closer (less than 5 Å) to Leu-446′ (Tyr-445′ in *Ba*IMPDH). Furthermore, the sugar moiety now also interacts with the protein. Specifically, the O2B ribose hydroxyl forms a hydrogen bond with the side chain of Ser-256, and ribose O4B interacts with the side chain Ser-251.

Two cofactor-bound *Vc*IMPDHΔL structures show that while maintaining the folded conformation, the adenosine moiety can exist in two orientations, one associated with the dehydrogenase and the other with the hydrolase step of the catalytic process. Interestingly, in both alternative conformations, the adenosine moiety makes contacts with several conserved residues from neighboring subunits. These include Leu-26′, Pro-27′, Ser-442′, Ser-256, and His-253 (Fig. 9*A*). Importantly, His-253 is capable of both a face-to-face and an edge-to-face type of interaction with the adenine ring. This may suggest a potential role of His-253 in dismissing the cofactor from the active site. All of these residues (except for Ser-442′, which is Ala-441′ in *Ba*IMPDH) are conserved in *Cp*IMPDH, *Ba*IMPDH, *Cj*IMPDH, and *Clp*IMPDH as well as in many other bacterial enzymes, suggesting that the cofactor binds in the same mode in all these proteins. Interestingly, *Vc*IMPDH contains Leu-446′ at the position where the four other bacterial IMPDHs contain the IMSM tyrosine (Tyr-′ in *Ba*IMPDH). This tyrosine forms a π/π-type interaction with the inhibitors (see above) and is likely to have a similar interaction with the adenine ring of the cofactor. Thus, the conservation of the inhibitor-binding site can now be explained by the presence of the new A-subsite in bacterial enzymes.

## DISCUSSION

Bacterial IMPDHs bind NAD^+^ with lower affinity than eukaryotic enzymes. The high and medium resolution structures of the cofactor complexes of *Vc*IMPDHΔL reveal a novel cofactor binding mode that differs dramatically from the one found in eukaryotic IMPDH structures both in the location of the A-subsite and in the conformations of the cofactor. This may account for the difference in affinity between bacterial and human IMPDHs. Thus, IMPDHs can be divided into two subclasses, one containing a eukaryote-like A^E^-subsite and the other containing a bacterium-like A^B^-subsite. In the A^E^-subsite, the adenine stacks between aromatic residues in the same monomer as the N-subsite, and the cofactor binds in a commonly observed extended conformation (Figs. 1*B* and 9*B*). In contrast, in bacterial IMPDHs the aromatic residues of the A^E^-subsite are replaced with small residues (Thr-230/Gly-259 in *Ba*IMPDH). These residues are not capable of stacking with adenine. Instead, the A^B^-subsite is relocated to the adjacent monomer where the adenine moiety interacts with a different set of residues localized at the interface between the monomers (His-254, Ser-257, Leu-26′, Pro-27′, Ala-441′, and Tyr-445′ in *Ba*IMPDH), and the cofactor assumes more compact conformations (Figs. 7–9, *A* and *C*).

Although the overall conformation of the cofactor is maintained in both *Vc*IMPDHΔL structures, the orientation of the adenosine moiety varies, suggesting that adenine binding is poor. In the structure with the mixture of a substrate and a covalent intermediate (*Vc*IMPDHΔL·IMP·NAD^+^), the adenosine sits deeper in the A^B^-subsite, and the cofactor is in a more compact conformation. This orientation may be necessary for the dehydrogenase step of the reaction. In the structure with the product (*Vc*IMPDHΔL·XMP·NAD^+^), the adenosine portion shifts toward the entrance to the A^B^-subsite, and the cofactor conformation, while still compact, becomes more open (Fig. 9*A*). This may indicate that the cofactor is withdrawing from the active site to make room for the hydrolysis stage of the reaction. Unfortunately, the active site flap, which contains the conserved arginine (Arg-403 in *Vc*IMPDH and Arg-404 in *Ba*IMPDH) implicated as a general base in hydrolysis, is partially disordered in all structures, and the arginine residue is not visible. The only clue about possible flap conformation during hydrolysis comes from the structure of the apo-form of *Ba*IMPDH (11).

Interestingly, the A^B^-subsite closely resembles the A-subsite of the related enzyme GMP reductase (GMPR (51)). This enzyme catalyzes the NADP^+^-dependent oxidation of IMP to E-XMP*, which reacts with ammonia to form GMP in GMPR. Unlike IMPDH, the cofactor is present during both the hydride transfer and amination steps of the GMPR reaction. Where the flap moves in IMPDH, the cofactor undergoes a conformational change in GMPR (51). These observations suggest that the catalytic cycle of bacterial IMPDHs, like the A-subsite, may have diverged between eukaryotic and bacterial IMPDHs.

Importantly, ascertaining the A^B^-subsite helps to rationalize the binding of the *Cp*IMPDH-selective inhibitors. The inhibitor closely resembles NAD^+^ (Fig. 9*D*), with one aromatic moiety interacting with the hypoxanthine ring of IMP in the N-subsite and the second aromatic ring interacting with the IMSM tyrosine residue in the A^B^-subsite. All of the inhibitor linkers are anchored via hydrogen bonds to Glu-416 (*Ba*IMPDH numbering). This glutamate is conserved among bacterial IMPDHs, yet it does not directly interact with NAD^+^. Glu-416 does form a hydrogen bond to the active site flap in the closed conformation required for the hydrolysis of E-XMP*. The IMSM alanine residue is part of the P-subsite, but the inhibitor linker binds in a very different manner than the pyrophosphate of NAD^+^. The variability of the P-subsite contrasts with other dehydrogenases where the pyrophosphate-binding motifs are not very specialized and seem to be designed to neutralize the negative charges (52). This observation also suggests that the P-subsite in IMPDH may not be as promiscuous as previously thought (6).

The significance of interactions within the P- and A^B^-subsites is clear in view of the *Vc*IMPDH lack of sensitivity to *Cp*IMPDH inhibitors. *Vc*IMPDH as well as IMPDH from *Escherichia coli* (*Ec*IMPDH) have a serine in place of the IMSM alanine. This serine maybe too large to accommodate the inhibitor and/or may disturb the inhibitor linker interactions with the conserved glutamate (Glu-416 in *Vc*IMPDH). Moreover, in both enzymes the IMSM tyrosine is replaced with a leucine residue (Leu-446 in *Vc*IMPDH) that is not capable of stacking with the aromatic moiety of the inhibitor (Fig. 2). Thus, because two sets of important contacts are removed, the affinity of the inhibitors for *Vc*IMPDH and *Ec*IMPDH (5) is at least 3 orders of magnitude lower. The lack of interactions between the IMSM tyrosine and the cofactor adenosine ring may also explain why *Vc*IMPDH and *Ec*IMPDH show lower affinity for NAD^+^ than the other three bacterial enzymes (Table 6) (4). Interestingly, when the corresponding residues in *E. coli* are mutated (S250A/L444Y) to restore the IMSM unit, the resulting double mutant becomes sensitive to the inhibitors (5).

Our inhibitor complexes provide important insights into the interactions that modulate selectivity and potency. In the N-subsite, higher affinity is observed with increased interactions with the hypoxanthine moiety of IMP. This can be achieved by adding ring substituents that expand the π/π contacts such as the sp2 centers in P-class compounds. These interactions also explain the low affinity of sp3 centers at this position (30). Within the P-subsite, as discussed above, potency is influenced by the hydrogen bond between the linker of the inhibitor and Glu-416 (*Ba*IMPDH numbering). This may explain the increased effectiveness of the urea-based linkers of P-class compounds that can form two hydrogen bonds with Glu-416. This residue is a glutamine in eukaryotic IMPDHs, and therefore it may also contribute to inhibitor selectivity. Within the A^B^-subsite, each inhibitor interacts with a set of residues involved in binding of the NAD^+^ adenine moiety, but additional inhibitor-specific contacts are important for increasing potency. These include halogen bonds between the 4-halogen substituents of **1**, **5**, **6** and the main chain carbonyl oxygen of bacterially conserved glycine residue (Gly-444 in *Ba*IMPDH) as well as water-mediated hydrogen-bonding interactions between the protein and the pyridine substituent of **7**. In addition to these features, our structures indicate that a further increase in potency might be achieved by mimicking the interactions of sugar and pyrophosphate groups of NAD^+^.

Significant differences are observed in the structure-activity relationships for inhibition of *Ba*IMPDHΔL, *Cj*IMPDHΔS, *Clp*IMPDHΔL, and *Cp*IMPDH despite the general conservation of their binding sites. For example, *Clp*IMPDHΔL has 10 times lower affinity for **2** than *Ba*IMPDHΔL and *Cj*IMPDHΔL (Table 7), even though the inhibitor interacts with the same residues in all three enzymes. Although our structures are of medium resolution (with the exception of 1.62 Å for *Vc*IMPDHΔL·XMP·NAD^+^) and thus are associated with a particular level of uncertainty, every interaction between *Clp*IMPDHΔL and **2** appears longer, and thus weaker, than the corresponding interaction in the other two enzymes. Because these variations in protein-inhibitor distances are small, it is clear that differences in inhibitor affinities must also derive from substitutions/factors outside the inhibitor-binding site. It is important to mention that the active site flap is partially disordered in our structures, and may possibly transiently interact with the inhibitor. Consequently, interactions with the flap may also determine inhibitor affinity and selectivity. We suggest that there are two different levels at which enzymes modulate selectivity and affinity toward ligands. Well defined ligand interactions with conserved active site residues provide the first level. Disruption of these interactions leads to a significant loss of binding and can alter ligand preference. The second level involves long distance effects (substitutions of residues away from the active site pocket, deletion of an entire domain, and change in active site flap sequence). These modifications may contribute to small structural changes or impact enzyme flexibility but are much more difficult to predict. However, these changes are important, as they allow fine-tuning affinity and selectivity.

This work shows that a different binding mode exists for the cofactor adenine moiety in bacterial IMPDHs. This finding opens new avenues for drug discovery. Our structures of bacterial IMPDHs complexes provide the basis for exploring inhibitor selectivity and offer a potential strategy for further ligand optimization that can be used to design more potent and selective inhibitors of bacterial IMPDHs.

## Footnotes

3 The abbreviations used are:

IMPDH: IMP dehydrogenase
*Ba*IMPDH: IMPDH from *Bacillus anthracis*
*Cp*IMPDH: IMPDH from *Cryptosporidium parvum*
*Clp*IMPDH: IMPDH from *Clostridium perfringens*
CBS: cystathionine β-synthetase
CPR: 6-chloropurine riboside 5′-monophosphate
*Ec*IMPDH: IMPDH from *Escherichia coli*
GMPR: GMP reductase
hIMPDH2: human IMPDH type 2
IMSM: inhibitor minimal structural motif
*Tf*IMPDH: IMPDH from *Tritrichomonas foetus*
PDB: Protein Data Bank
IMAC: immobilized metal affinity chromatography
*Vc*IMPDH: IMPDH from *V. cholerae*.
